# *Moniliella spathulata*, an oil-degrading yeast, which promotes growth of barley in oil-polluted soil

**DOI:** 10.1007/s00253-020-11011-1

**Published:** 2020-11-20

**Authors:** Annett Mikolasch, Ramza Berzhanova, Anel Omirbekova, Anne Reinhard, Daniele Zühlke, Mareike Meister, Togzhan Mukasheva, Katharina Riedel, Tim Urich, Frieder Schauer

**Affiliations:** 1 Institute of Microbiology, University Greifswald, Felix-Hausdorff-Straße 8, 17489 Greifswald, Germany; 2 Department of Biology and Biotechnology, Al-Farabi Kazakh National University, Al-Farabi Ave 71, Almaty, Kazakhstan 050040; 3 Leibniz Institute for Plasma Science and Technology (INP), Felix-Hausdorff-Str. 2, 17489 Greifswald, Germany

**Keywords:** *Moniliella spathulata*, *Trichosporonoides spathulata*, Crude oil, Biphenyl, Dibenzofuran

## Abstract

**Abstract:**

The yeast strain *Moniliella spathulata* SBUG-Y 2180 was isolated from oil-contaminated soil at the Tengiz oil field in the Atyrau region of Kazakhstan on the basis of its unique ability to use crude oil and its components as the sole carbon and energy source. This yeast used a large number of hydrocarbons as substrates (more than 150), including *n*-alkanes with chain lengths ranging from C_10_ to C_32_, monomethyl- and monoethyl-substituted alkanes (C_9_–C_23_), and *n*-alkylcyclo alkanes with alkyl chain lengths from 3 to 24 carbon atoms as well as substituted monoaromatic and diaromatic hydrocarbons. Metabolism of this huge range of hydrocarbon substrates produced a very large number of aliphatic, alicyclic, and aromatic acids. Fifty-one of these were identified by GC/MS analyses. This is the first report of the degradation and formation of such a large number of compounds by a yeast. Inoculation of barley seeds with *M. spathulata* SBUG-Y 2180 had a positive effect on shoot and root development of plants grown in oil-contaminated sand, pointing toward potential applications of the yeast in bioremediation of polluted soils.

**Key points:**

• *Moniliella spathulata an oil-degrading yeast*

• *Increase of the growth of barley*

**Supplementary Information:**

The online version contains supplementary material available at 10.1007/s00253-020-11011-1.

## Introduction

The yeast SBUG-Y 2180 was isolated from a sample of oil-contaminated soil from the Tengiz oil field in the Atyrau region of Kazakhstan. The Tengiz oil reservoir was discovered in 1979. It is approximately 21 km long and 19 km wide and is one of the largest oil fields worldwide with reserves estimated at between six billion and nine billion barrels (GlobalData.com 2020; Pala 2001). It is considered to be the largest single-trap producing reservoir in existence (Chevron.com 2020). There have been a number of accidental spills during the exploitation of this deposit, because the oil is hot and under greater pressure than has been seen at any other location. In addition, the oil contains a large proportion of gas, and is rich in H_2_S. These properties of the Tengiz reservoir are serious challenges for its exploitation, even when the latest oil production technology is used. The most dangerous accident was an explosion in 1985, which produced a 200-m-high fire column that burned for more than a year (Pala 2001). This and other accidents have led to the pollution of the surrounding soil.

Many attempts have been made worldwide to use biological systems for bioremediation of oil-polluted soil (Dua et al. 2002; Ekperusi and Aigbodion 2015; Juwarkar et al. 2010; Margesin and Schinner 2001). Among the various methods available, the combination of oil-consuming prokaryotic strains, together with plants growing in the contaminated areas, can result in the efficient cleaning of polluted soils. The microbial communities cooperate metabolically and exchange metabolites and end products between each other and with plants in the neighborhood (Ivanova et al. 2015; Kuiper et al. 2004; Mikolasch et al. 2016).

Till now, bacterial or fungal (Russo et al. 2019) strains have been usually applied for bioremediation of polluted areas. Soil yeasts have not been used to the same extent because they tend to have a strong endemism and there are a surprisingly high number of currently unidentified species (Yurkov 2018). On the other hand, aliphatic hydrocarbons such as *n*-alkanes of different chain lengths—an important compound group in crude oil—were used by a great variety of yeasts as the sole source of carbon and energy (Bos and de Bruyn 1973; Schauer and Schauer 1986). Approximately 20% of all yeast strains are able to use hydrocarbons. The ability to degrade aliphatic and aromatic hydrocarbons involves well-studied reactions, which are widely available among the ascomycetous (e.g., *Debaryomyces*, *Exophiala*, *Lodderomyces*, *Metschnikowia*, *Pichia*, or *Yarrowia*), the basidiomycetous (e.g., *Cryptococcus*, *Rhodosporidium*, or *Sporidiobolus*), and the imperfect species (e.g., *Candida*, *Rhodotorula*, or *Trichosporon*) (Bos and de Bruyn 1973; Middelhoven 1993; Watkinson and Morgan 1990).

The aims of the current study were to investigate the oil-degrading yeast SBUG-Y 2180 taxonomically, to explore its potential to degrade oil components, and to analyze its ability to promote plant growth on oil-polluted soil.

## Materials and methods

### Enrichment and isolation of the yeast SBUG-Y 2180

The yeast SBUG-Y 2180 was enriched from a contaminated soil sample of the Tengiz oil field in the Atyrau region of Kazakhstan on Tengiz crude oil–containing media according to the method of Joo et al. (2008), and isolated by plating 0.1 mL of the enriched cultures on Sabouraud agar (Merck, Germany). Pure cultures were cultivated on malt agar slants.

### Identification of B1

Physiological tests—urease test (Seeliger 1956) and 32 miniaturized assimilation tests with the API 32 C test kit (biomerieux, Germany)—were performed using standard methods. The yeast was grown, and cells were then opened for DNA isolation as described (Mikolasch et al. 2019), method 3), and characterized by ITS gene sequence analyses (Mikolasch et al. 2019). Yeast almost full-length ITS genes were amplified using 1 μL DNA extract (cell material of one colony in 20 μL ddH_2_O) as template with oligonucleotides ITS1 (TCCGTAGGTGAACCTGCGG, 0.5 μM) and ITS4 (TCCTCCGCTTATTGATATGC, 0.5 μM) (White et al. 1990) as primers. Sanger sequencing was performed by Eurofins Genomics (Germany) with ITS1 and ITS4 primers, respectively. The resulting forward and reverse sequences were assembled using the program Geneious (geneious, USA). The almost full-length ITS sequence was compared with the NCBI nr database using the blastn algorithm (Altschul et al. 1990) and the mycobank (*Westerdijk Fungal Biodiversity Institute*, *Utrecht*, *The Netherlands).* The best hit of the NCBI nr database and mycobank alignment—*Moniliella spathulata* strain CBS 241.79—was purchased from the Westerdijk Fungal Biodiversity Institute (CSB), and was treated by the same methods as the cell material of the yeast SBUG-Y 2180. The ITS sequences of the yeast SBUG-Y 2180 and the CBS *Moniliella spathulata* strain CBS 241.79 were compared using the blastn algorithm.

### Evaluation of growth in the presence of different oils

For growth tests on different oils in liquid medium, the yeast SBUG-Y 2180 was cultivated in 500-mL flasks containing 100 mL of mineral salt medium for fungi (MSMF) pH 5.4 supplemented with 1% vitamin solution (Awe et al. 2008) and with 3% [v/v] oil as single substrate at 30 °C and 130 rpm. After incubation, the cells were harvested, dried, and analyzed according the dry weight method (Mikolasch et al. 2015).

### Evaluation of growth in the presence of biphenyl and dibenzofuran

For growth tests on the aromatic compounds biphenyl and dibenzofuran, SBUG-Y 2180 was cultivated on solid medium MSMF supplemented with 1% vitamin solution and with 20 mg L^−1^ biphenyl or dibenzofuran as single substrate at 30 °C.

### Degradation experiments

#### Crude oil as single substrate

Cultures of SBUG-Y 2180 were shaken in 500-mL flasks with 100 mL MSMF medium, 1% vitamin solution, and 1 mL crude oil (Uzen deposit, Mangystau region, Kazakhstan) at 30 °C and 180 rpm for 7, 14, and 28 days. Assays without oil or without cells or with cells and 1% glucose as sole source of carbon and energy were used as controls. All controls were treated as the transformation assays.

The data are presented as the average of four separate experiments with replicated batch cultures. These replicates did not have standard deviations more than 10%.

#### Pristane and tetradecane as substrate mixture

Cells were pre-grown on MSMF plates (Awe et al. 2008; Mikolasch et al. 2019) with 0.4 mL tetradecane as substrate on a filter paper in the lid of the plates. Pre-cultivated cells of the isolated strain were shaken in 500-mL flasks containing 100 mL MSMF plus 0.5 mL tetradecane and 0.5 mL pristane as carbon and energy sources at 30 °C and 180 rpm for 7 days. Assays without substrates and without cells were used as controls. Cell material from well-grown plates was used to prepare an inoculation suspension of 5 mL. In each case, 1 mL of this was used to inoculate the parallel transformation assays and the control flasks. All controls were treated as the transformation assays.

#### Undecylcyclohexane as single substrate

Cells were treated as for the degradation experiments with pristane and tetradecane, but using undecylcyclohexane (0.1% (v/v)) as substrate.

#### Biphenyl or dibenzofuran as single substrate

Ten milligrams of biphenyl (BP) or dibenzofuran (DBF) dissolved in diethyl ether was added to sterile 500-mL flasks. After evaporation of the diethyl ether for 24 h, 100 mL of MSMF were added to each flask, and the flasks were shaken for 24 h at 30 °C and 180 rpm to achieve saturation of the compounds in the liquid medium. A cell suspension with glucose-grown cells was then added to an optical density (*A* 600 nm) of 2.00. Cultures were incubated on a rotary shaker at 30 °C and 180 rpm for 7 days. Flasks with cell suspension in medium with 1% glucose as substrate, as well as flasks without substrate or flasks without cells, were used as controls. All controls were treated as the transformation assays.

### Identification of degradation products

After incubation on the various substrates, whole cultures (medium, oil, and cells) were subjected to alkaline and acidic extraction, and the extracts were analyzed by GC/MS as described previously (Mikolasch et al. 2019; Mikolasch et al. 2015). Supernatants of cultures incubated on BP or DBF were analyzed by HPLC-UV/Vis (Awe et al. 2008) and after extraction (Mikolasch et al. 2019; Mikolasch et al. 2015) by GC/MS.

### Protein extraction and mass spectrometry analyses

Cells were grown in triplicates for 14 days in 100 mL of MSMF medium with glucose or oil as carbon source. Thirty-milliliter cultures were harvested by centrifugation (10 min, 4 °C, 8500 rpm), washed three times with 2 mL TE buffer, and finally resuspended in 1 mL TE buffer. Cell suspensions were transferred into screw cap tubes filled with 500 μL of lysis matrix A (MP Biomedicals) and mechanically disrupted using a FastPrep (MP Biomedicals) for 3 × 30 s at 6.5 m/s with on-ice incubation for 5 min between cycles. Cell debris was removed by centrifugation (5 min, 4 °C, 13,000 rpm), followed by a second centrifugation step (30 min, 4 °C, 13,000). Protein concentration was determined with the Pierce BCA Protein Assay Kit (Thermo Scientific). Thirty micrograms of protein extract was separated on a 1D SDS PAGE and gel lanes cut into 5 (glucose samples) or 1 (oil samples) fractions. Tryptic in-gel digestion was described earlier (Eymann et al. 2017). Resulting peptide mixes were desalted using C_18_ Zip Tips (Thermo Scientific).

LC-MS/MS analyses were done on an EASY-nLC1200 coupled to an QExactive HF mass spectrometer (Thermo Fisher Scientific). Peptides were separated on a self-packed analytical column (100 μm × 20 cm) containing reverse-phase C_18_ material with an integrated emitter using an 85-min non-linear gradient from 5 to 50% buffer B (0.1% acetic acid in acetonitrile) and a flow rate of 300 nl/min. Survey scans were recorded in the Orbitrap with a resolution of 60,000 in the m/z range of 333–1650. The 15 most-intense peaks per scan cycle were selected for fragmentation. Precursor ions were dynamically excluded from fragmentation for 30 s; singly charged ions as well as ions with unknown charge state were rejected. Internal calibration was enabled (lock mass 445.12003).

Since the genome sequence of *Moniliella spathulata* is not available yet, the sequence of *Moniliella* sp. ‘wahieum’ (NCBI; assembly ASM397190v1) was used to identify proteins. The genome sequence of *Moniliella* sp. ‘wahieum’ was uploaded to the Galaxy web platform, and *the public server at* usegalaxy.org*was used to analyze the sequence (*Afgan et al. 2018*)*. Gene prediction and translation into protein sequences were done using Augustus (Galaxy version 3.3.3) (Stanke et al. 2008). Functional annotation of predicted proteins was done using eggNOG-mapper (v2.0.0) (Huerta-Cepas et al. 2017; Huerta-Cepas et al. 2019). Database searching and quantification were performed using MaxQuant software (v6.1.10.43) (Cox and Mann 2008). MS and MS/MS spectra were searched against the *Moniliella* sp. ‘wahieum’ database (29,737 entries) using the following parameters: protease trypsin, two missed cleavages allowed, variable modification methionine oxidation, precursor ion mass tolerance 20 ppm, and fragment ion mass tolerance 0.5 Da. Mass spectrometry proteomics data have been deposited to the ProteomeXchange Consortium via the PRIDE partner repository (Perez-Riverol et al. 2019) with the dataset identifier PXD022543.

### Microbial inoculation of barley seeds by the yeast SBUG-Y 2180

The ability of the yeast SBUG-Y 2180 to support the growth of barley on oil-contaminated soil was tested by the barley seed inoculation method (Mikolasch et al. 2015) using an incubation temperature of 28 °C for 7 days.

## Results

### Identification of the yeast SBUG-Y 2180

**Fig. 1:**
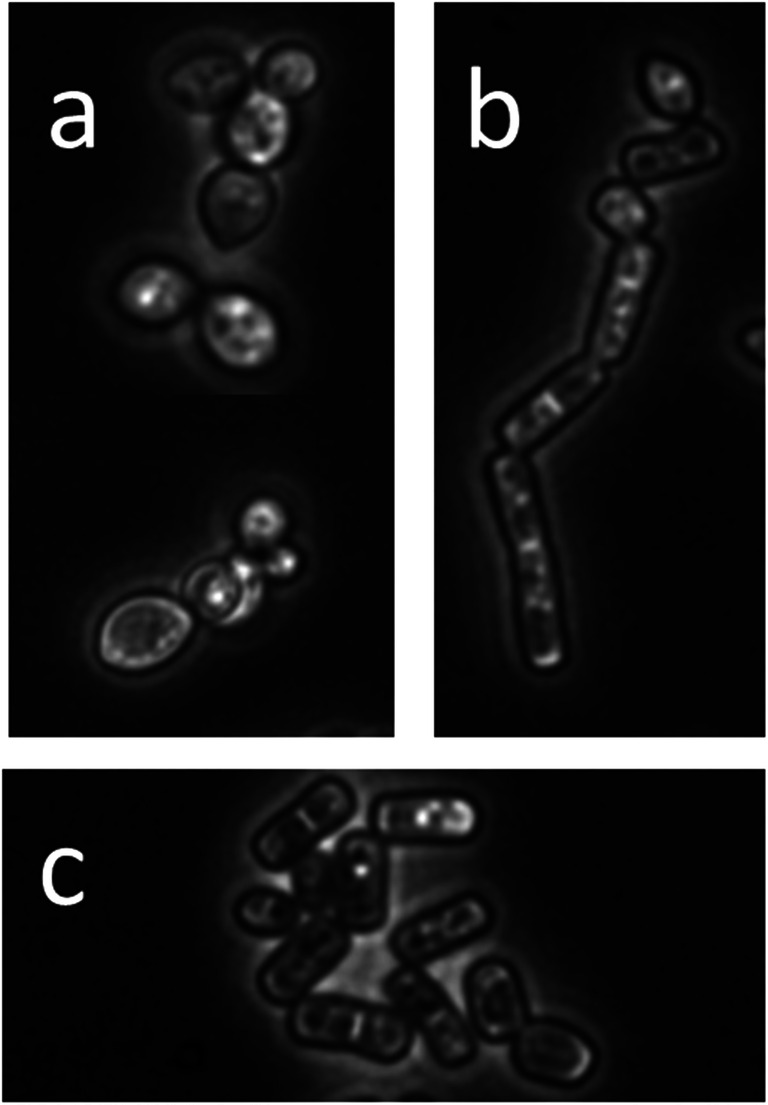
**a** Yeast-like cells with multilateral budding, **b** hyphae with acropetal sprouting blastokonidia, **c** arthroconidia of the yeast SBUG-Y 2180; 40-fold microscopic magnification

The yeast SBUG-Y 2180 was characterized by colony appearance and cell morphology; by urease test with Christensen’s medium; by sugar, cycloheximide, and glucosamine assimilation tests with the API 32 C test kit; and by ITS gene sequence analyses. The isolated strain SBUG-Y 2180 produced large olive pigmented colonies with a diffuse border and a “hill” in the middle. When grown on malt agar plates, yeast-like cells with multilateral budding (Fig. 1a), hyphae with existing acropetal sprouting blastokonidia (Fig. 1b), and arthroconidia (Fig. 1c) were observed, as described for *Moniliella spathulata* (Kurtzman et al. 2011; Rosa et al. 2009). Formation of chlamydospores was not observed.

A positive urease test identifies the yeast as a basidiomycete. Furthermore, comparison of the SBUG-Y 2180 ITS gene sequence analyses with the NCBI nr database and the mycobank showed the highest sequence identity with *Moniliella spathulata* CBS 241.79 (Supplementary Table S1 and S2). When cells of this strain, obtained from the CSB strain collection, were treated by the same methods as the yeast SBUG-Y 2180 cells, they yielded similar results (Supplementary Table S3 and S4). Sequences of SBUG-Y 2180 cells and CBS 241.79 cells were compared using the blastn algorithm and showed sequence identity (100%) using the ITS1 sequences and sequence similarity of 99.65% using the assembled ITS sequences (Supplementary Table S5 and S6). Furthermore both, CBS 241.79, which is the type sample defining the species *Moniliella spathulata* (Kurtzman et al. 2011), and SBUG-Y 2180 were used for assimilation tests (sugars, cycloheximide and glucosamines) with the API 32 C test kit. The results of all 32 miniaturized assimilation tests of SBUG-Y 2180 were the same as for CBS 241.79 material (Supplementary Table S7), though there were three differences to the data given in the original description (Kurtzman et al. 2011). The isolated and identified yeast SBUG-Y 2180 was deposited at the strain collection of the Department of Biology of the University Greifswald (SBUG) and named *Moniliella spathulata* SBUG-Y 2180 (B1). The ITS sequence was deposited in GenBank: *Moniliella spathulata* SBUG-Y 2180 (B1) Accession No. MT003285.

### Growth characteristics and crude oil consumption of *Moniliella spathulata* SBUG-Y 2180

Different types of oil were used by *M. spathulata* SBUG-Y 2180 as the sole source of carbon and energy. The dry weight increased during cultivation in the mineral salt medium on crude oil, diesel fuel, and fuel oil for 6 days, and continued to increase during cultivation for a further 4 days (Table 1). After 10 days, *M. spathulata* SBUG-Y 2180 had grown well on crude oil with 72% of the growth on Sabouraud medium, whereas the growth on diesel fuel and fuel oil was much reduced, to 21% and 36%, respectively.

**Table 1:** Growth experiments with *M. spathulata* SBUG-Y 2180 on 3% [v/v] of different oils after cultivation for 6 and 10 days and controls

Dry weight after growth on different substrates [g L^−1^]		
Substrate	6 days	10 days
Crude oil	5.6 (0.054)^a^	6.9 (0.034)
Diesel fuel	2.8 (0.005)	3.5 (0.033)
Fuel oil	1.4 (0.008)	2.0 (0.005)
Sabouraud medium	8.3 (0.087)	9.6 (0.217)
Dry weight of innoculum 0.03 (0.001) [g L^−1^]		

^a^Standard deviation

As crude oil was the best substrate for growth, *M. spathulata* SBUG-Y 2180 was incubated with this oil as the sole carbon source, for a detailed investigation of the consumption of different oil components. After 7, 14, and 28 days of cultivation, whole cultures were extracted and residues were dissolved and analyzed by GC/MS (Supplementary Table S8).

The volatile *n*-alkanes with C_9_–C_12_ chain length and *n*-alkylcyclohexanes with chain length from C_2_–C_4_ evaporated completely or in part during the incubation process and could therefore not be analyzed by GC/MS (Mikolasch et al. 2015).

The other *n*-alkanes with chain length from C_12_ to C_32_ were completely degraded within 7 days of incubation. Most of the other oil components detectable by GC/MS such as alkylcyclohexanes, branched-chain alkanes, and aromatics were not fully degraded, even after 28 days. However, most of the *n*-alkylcyclohexanes, the branched-chain alkanes, the alkylmono- and diaromatics, and the alkylnaphthalenes were transformed to varying degrees during incubation and even aromatics without alkyl side chains like biphenyl and naphthalene were degraded by 50% and 100%, respectively.

To verify the transforming potential and to characterize the degradation pathways of the crude oil components, cultures of *M. spathulata* SBUG-Y 2180 grown on oil were extracted at pH 2 and extracts were methylated using diazomethane and analyzed by GC/MS. A large number of acidic products were identified by comparison with standard compounds or with the spectral library of the National Institute of Standard Technology (NIST08; overview Table 2; mass spectrum data Supplementary Tables S9–S14). Sixteen different *n*-alkyl- and branched-chain alkyl-substituted monocarboxylic acids (M1–M16), 8 *n*-alkyl- and branched-chain alkyl-substituted dicarboxylic acids (M17–M24), 5 cyclohexylalkanoic acids (M25–M29), 3 cyclopentylalkanoic acids (M30–M32), 14 phenylalkanoic acids (M33–M46), 3 naphthylalkanoic acids (M47–M49), and 2 biphenylalkanoic acids (M50 and M51) were detected. None of these were present in the original oil sample. In summary, 51 different acidic products were identified as transformation products of oil components produced by *M. spathulata* SBUG-Y 2180 during incubation on crude oil. In control measurements of the assays with glucose as sole source of carbon and with crude oil without cells, no acidic products were detected.

**Table 2:** Aliphatic and aromatic parent components of crude oil and the acids formed by *M. spathulata* SBUG-Y 2180 detected by GC/MS during growth on crude oil

Detected oil components		Detected transformation products^a^	
Name	Transformation extent^b^	Number	Name
*n*-Alkanes (23 detected compounds)			
Decane to dotriacontane	Total	3	Heptanoic acid M7^c^ Hexanedioic acid M19 Decanedioic acid M24
Branched-chain alkanes (numerous)			
Monoethyl-substituted alkanes	Total	2	2-Ethyl-hexanoic acid M10 2-Ethylidene-propanedioic acid M17
Monomethyl-substituted alkanes	Total	13^d^	3-Methyl-but-2-enoic acid M1 3-Hydroxy-3-methyl-butanoic acid M3 3-Methyl-pentanoic acid M4 3-Methyl-pent-2-enoic acid M5 3-Methyl-2-oxo-pentanoic acid M6 2-Hydroxy-3-methyl-but-2-enoic acid M8 3-Methyl-hex-2-enoic acid M9 2-Hydroxy-4-methyl-pent-2-enoic acid M11 3-Methyl-pentanedioic acid M18 2-Methyl-hexanedioic acid M20 3-Methyl-hexanedioic acid M21 3-Methyl-heptanedioic acid M22 4-Methyl-heptanedioic acid M23
Polymethyl-substituted alkanes e.g., 2,6,10-Trimethyl-dodecane 2,6,10-Trimethyl-pentadecane Pristane (2,6,10,14-tetramethylpentadecane) 2,6,10,14-Tetramethylhexadecane	Partial		
		5 ^*e)*^	2,3-Dimethyl-butanoic acid M2 Dimethyl-octanoic acids M13, M14, M15, M16
Alkylcyclohexanes			
*n*-Alkylcyclohexanes (23 detected compounds)			
Ethylcyclohexane to octadecylcyclohexane	Partial	4	Cyclohexanecarboxylic acid M25 1-Cyclohexene-1-carboxylic acid M27 Cyclohexylacetic acid M28 Cyclohexylpropanoic acid M29
*n*-Alkyl-methylcyclohexanes (40 detected compounds)			
Methyl-substituted	Total	1	4-Methyl-cyclohexane-1-carboxylic acid M26
Alkylcyclopentanes			
*n*-Alkylcyclopentanes (compounds detected in traces)			
*n*-Alkylcyclopentanes	Total	2	Cyclopentanecarboxylic acid M30 1-Cyclopentene-1-carboxylic acid M31
*n*-Alkyl-methylcyclopentanes (compounds detected in traces)			
Methyl-substituted	Total	1	3-Methyl-cyclopentane-1-carboxylic acid M32
Alkylbenzenes (28 well-detected, and traces of longer-chain compounds)			
*n*-Alkyl-substituted benzenes	Partial	5	Benzoic acid M33 Phenylacetic acid M34 2-Hydroxy-, 4-hydroxy-benzoic acid M35, M44 3,4-Dihydroxy-benzoic acid M46
Poly-*n*-alkyl-substituted benzenes	Total	6	3-Methyl, 4-methyl-benzoic acid M36, M37 4-Methyl-phenylacetic acid M38 2,4-Dimethyl, 3,5-dimethyl-benzoic acid M40, M42 4-Ethyl-benzoic acid M41
Branched-chain-alkyl-substituted benzenes	Total	3	2-Phenylpropionic acid M39 2-Phenylbutyric acid M43 3-Phenylbutyric acid M45
Naphthalenes (4 well-detected, and traces of longer-chain compounds)			
Naphthalene	Total	–	
*n*-Alkyl-substituted naphthalenes	Partial	3	1- and 2-naphthalenecarboxylic acid M47, M48 1-Naphthaleneacetic acid M49
Biphenyls (3 well-detected, and traces of longer-chain compounds)			
Biphenyl	Partial	–	
*n*-Alkyl-substituted biphenyls	Partial	2	4-Biphenylcarboxylic acid M50 4-Biphenylacetic acid M51

^a^See structures and analytical data of the acids formed in Supplementary Tables S9–S14

^b^See the amount of transformation [%] after 7, 14, and 28 days in Supplementary Table S8

^c^Names of acidic products with numbers according to increasing retention times in the GC elution profile, Supplementary Tables S9–S14

^d^Possible detected transformation products of mono- and polymethyl-substituted alkanes

^e^Possible detected transformation products of polymethyl-substituted alkanes only

More than 150 different components of crude oil can be converted by *M. spathulata* SBUG-Y 2180. Furthermore, the oil components pristane, biphenyl, and dibenzofuran, compounds that are otherwise difficult to metabolize, were also utilized as sole sources of carbon and energy in degradation experiments with *M. spathulata* SBUG-Y 2180.

### Biodegradation of pristane

Pristane and tetradecane were used as a substrate mixture to induce the alkane-degrading enzymes necessary for the biodegradation of pristane. After 14 days, five acidic transformation products of pristane were detected—a branched-chain alkyl-substituted monocarboxylic acid (pristanic acid MP4) and four branched-chain alkyl-substituted dicarboxylic acids (MP1, MP2, MP3, and MP5; Supplementary Table S15).

### Consumption of biphenyl and dibenzofuran

BP or DBF were used as single sources of carbon and energy in liquid MSMF over a period of 14 days (Table 3). *M. spathulata* SBUG-Y 2180 was able to grow on, and to transform, both substrates. The monohydroxylated biphenyls 3- and 4-hydroxybiphenyl and all possible monohydroxylated dibenzofurans 1-, 2-, 3-, and 4-hydroxydibenzofuran were identified (Supplementary Table S16). Ring cleavage products were not detected.

**Table 3:** Growth of *M. spathulata* SBUG-Y 2180 on biphenyl and dibenzofuran and metabolites formed by *M. spathulata* SBUG-Y 2180 during transformation of biphenyl and dibenzofuran

Biphenyl			
Biphenyl growth		Control growth on glucose	
7 days	14 days	7 days	14 days
++	+++	+++	+++
Biphenyl metabolites after 14 days			
4-OH-BP (MBP1)	3-OH-BP (MBP2)	2-OH-BP	
d	d	nd	
Dibenzofuran			
Dibenzofuran growth		Control growth on glucose	
7 days	14 days	7 days	14 days
+	++	+++	+++
Dibenzofuran metabolites after 14 days			
4-OH-DBF (MDBF1)	3-OH-DBF (MDBF2)	2-OH-DBF (MDBF3)	1-OH-DBF (MDBF4)
d	d	d	d

++ good growth, +++ very good growth, *d* detected and identified using HPLC and/or GC/MS (Supplementary Table S16), *nd* not detected using HPLC and/or GC/MS (Supplementary Table S16)

### Transformation of phenylalkanes

Undecylcyclohexane was used as a single substrate and as a model for phenylalkane transformation by *M. spathulata* SBUG-Y 2180. The aim of these experiments was to determine whether the cyclohexylalkanoic acids cyclohexanecarboxylic acid M25, 1-cyclohexene-1-carboxylic acid M27, and cyclohexylacetic acid M28 (Supplementary Table S11) were end products of the transformation of the phenylalkanes of crude oil, or whether they were metabolized further. After 14 days of incubation, five acidic transformation products of undecylcyclohexane were identified. In addition to the cyclohexylalkanoic acids M25, M27, and M28 (now named MC_6_C_11_1, MC_6_C_11_3, and MC_6_C_11_4 in Supplementary Table S17), two aromatic acids—benzoic acid MC_6_C_11_2 and phenylacetic acid MC_6_C_11_5—were detected.

### Identification of candidate enzymes involved in transformation of crude oil compounds

Both *M. spathulata* SBUG-Y 2180 and the CBS *M. spathulata* strain CBS 241 were grown in the presence of glucose and crude oil, respectively. Only for glucose-grown cells was protein extraction successful; therefore, we were not able to identify enzymes that might be specifically synthesized in the presence of oil. Tryptic peptides of glucose-grown cells were analyzed by LC-MS/MS to identify possible candidates that might be involved in the degradation/transformation of crude oil. We could detect 24 proteins that might be associated with the transformation of oil according to their functional prediction (Table 4). This includes enzymes involved in aromatic ring cleavage (RSEE01000002.1.g1287), degradation of aliphatic carbohydrates (e.g., RSEE01000006.1.g3392, RSEE01000006.1.g3393, RSEE01000053.1.g18113, RSEE01000008.1.g4308, RSEE01000073.1.g21446, RSEE01000022.1.g10034, RSEE01000031.1.g12774, RSEE01000079.1.g22204, RSEE01000076.1.g21850, RSEE01000029.1.g12222), or initial hydroxylations (RSEE01000002.1.g906).

**Table 4:** Proteins identified by LC-MS/MS with possible involvement in oil degradation in *M. spathulata* CBS 241 and SBUG-Y 2180

Protein accession^a^	Description^b^	ID CBS 241^c^	ID SBUG-Y 2180^c^
RSEE01000002.1.g1287	Catalyzes the oxidative ring opening of 3-hydroxyanthranilate to 2-amino-3-carboxymuconate semialdehyde, which spontaneously cyclizes to quinolinate	X	X
RSEE01000002.1.g906	monooxygenase	X^*^	X
RSEE01000006.1.g3392	Aldehyde dehydrogenase family	X	X
RSEE01000006.1.g3393	Aldehyde dehydrogenase family	X	X
RSEE01000008.1.g4308	Phytanoyl-CoA dioxygenase (PhyH)	X	X
RSEE01000022.1.g10034	Acyl-CoA dehydrogenase, C-terminal domain	X	X
RSEE01000025.1.g11051	Alcohol dehydrogenase GroES-like domain	X	X
RSEE01000025.1.g11099	Belongs to the acyl-CoA oxidase family	X^*^	X
RSEE01000029.1.g12222	Belongs to the thiolase family	X	X
RSEE01000031.1.g12774	Alpha-beta-hydrolase	X	X
RSEE01000034.1.g13603	Dyp-type peroxidase family	X	X^*^
RSEE01000036.1.g14415	Belongs to the aldehyde dehydrogenase family	X	X
RSEE01000047.1.g16882	Belongs to the aldehyde dehydrogenase family	X^*^	–
RSEE01000052.1.g18003	Aldehyde dehydrogenase family	X	X
RSEE01000053.1.g18113	Belongs to the thiolase family	–	X^*^
RSEE01000056.1.g18812	Belongs to the aldehyde dehydrogenase family	–	X^*^
RSEE01000059.1.g19195	Catalyzes a 2-step reaction, involving the ATP-dependent carboxylation of the covalently attached biotin in the first step and the transfer of the carboxyl group to pyruvate in the second	X	X
RSEE01000073.1.g21446	D-Isomer specific 2-hydroxyacid dehydrogenase, catalytic domain	X	X
RSEE01000075.1.g21656	D-Isomer specific 2-hydroxyacid dehydrogenase, catalytic domain	X	X
RSEE01000076.1.g21850	3-Hydroxyacyl-CoA dehydrogenase, NAD binding domain	X	X
RSEE01000079.1.g22204	Alpha-beta-hydrolase	X	X
RSEE01000108.1.g25431	Belongs to the peroxidase family	X	X
RSEE01000159.1.g28641	Belongs to the aldehyde dehydrogenase family	X	X
RSEE01000187.1.g29162	Peroxidase	X	X

^a^Protein accessions were provided by the Galaxy web platform (see MM for details)

^b^Functional prediction is based on eggNOG-mapper

^c^Enzymes that were identified in the respective strain with at least two peptides are marked with “X”; additionally, if a protein was only identified in one out of three biological replicates, it is marked with “^*^”

### Influence of yeast inoculation of barley seeds on the plant development

All the described experiments showed that *M. spathulata* SBUG-Y 2180 is a highly potent yeast for crude oil degradation. In order to show that this yeast not only theoretically has a high degradation potential but actually has an influence on the growth of plants that are used for soil remediation of oil-contaminated areas, barley seeds and *M. spathulata* SBUG-Y 2180 cells were used together in soil experiments.

**Fig. 2:**
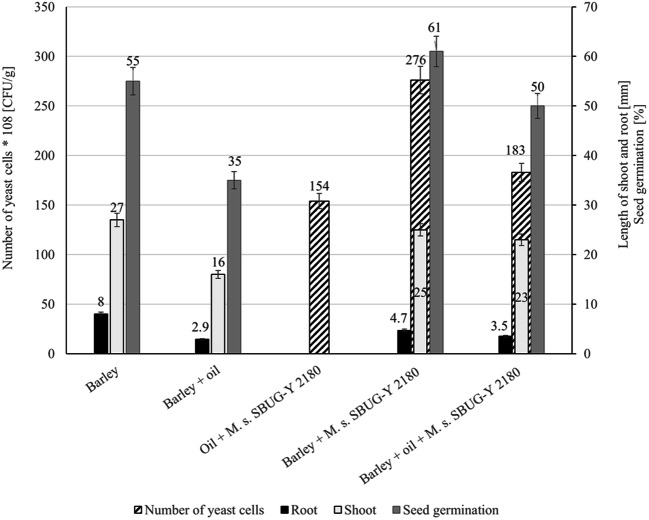
Influence of *M. spathulata* SBUG-Y 2180 inoculation of barley seeds on the plant development in oil-containing sand after 7 days. Number of yeast cells of *M. spathulata* SBUG-Y 2180 at the beginning: 25 × 10^6^ CFU/g sand

Barley seeds were inoculated with *M. spathulata* SBUG-Y 2180 at a cell density of 25 × 10^6^ CFU/g sand, and sown in oil-containing sand (2% oil). Plants were grown for 7 days and the number of CFU of *M. spathulata* SBUG-Y 2180, the lengths of shoots and roots, and the rate of germination were then measured (Fig. 2). A considerable protective effect on barley was observed in the growth assay inoculated with *M. spathulata* SBUG-Y 2180. This treatment of seeds stimulated the growth of barley seedlings in oil-contaminated sand, increasing the growth of shoots by 44% and of roots by 20% compared with the growth in oil-containing sand without inoculation. The P(T<-t) values are 1.06E−10 and 2.16E−06, respectively, and they support the hypothesis of significant growth increase by the inoculation of seeds with *M. spathulata* SBUG-Y 2180 in oil-containing sand. It should be emphasized that the root growth of barley seedlings inoculated with *M. spathulata* SBUG-Y 2180 in pristine sand is also 41% lower than in assays without inoculation and pristine sand. Nevertheless, there is better root growth in oil-contaminated sand with the yeast than without the yeast inoculation. Furthermore, the rate of seed germination increased by 15% and was only 5% lower than that of barley growth without oil. Parallel experiments with inoculation of barley seeds with *M. spathulata* SBUG-Y 2180 and their growth in sand without oil and experiments without barley were conducted. The inoculation of seeds with the yeast had no positive effect on growth of barley without oil. Furthermore, the number of CFU increased by a factor of 600 in the control assay with oil, but without plants. In comparison, the number of CFU of the assay with barley, *M. spathulata* SBUG-Y 2180, and oil was 19% higher and that of the assay with barley and *M. spathulata* SBUG-Y 2180 and without oil was 79% higher than that of the control assay. The P(T<-t) values are 2.65E−07 and 4.98E−13, respectively, and they show significantly higher CFUs and indicate that the presence of barley increases the development of the yeast. All results together show that *M. spathulata* SBUG-Y 2180 promotes growth and germination of barley in the oil-contaminated sand and barley also positively influences the development of *M. spathulata* SBUG-Y 2180.

## Discussion

The yeast strain SBUG-Y 2180 was isolated from oil-contaminated soil of the Tengiz oil field in the Atyrau region of Kazakhstan. This yeast was identified as the basidiomycete *Moniliella spathulata*. Although this species has not been previously isolated from oil-contaminated areas, other species of *Moniliella* have been isolated from such oil-rich habitats (Ching et al. 2016; Ye et al. 2017). *M. spathulata* was formerly known as *Trichosporonoides spathulata*, but in 2009, all species of *Trichosporonoides* were transferred to the genus *Moniliella* because both genera are members of a single monophyletic clade (Rosa et al. 2009). *T. spathulata* has been isolated from the waste of palm oil mills and biodiesel plants (Kitcha and Cheirsilp 2013), which can contain similar hydrocarbons as oil-contaminated habitats.

**Fig. 3:**
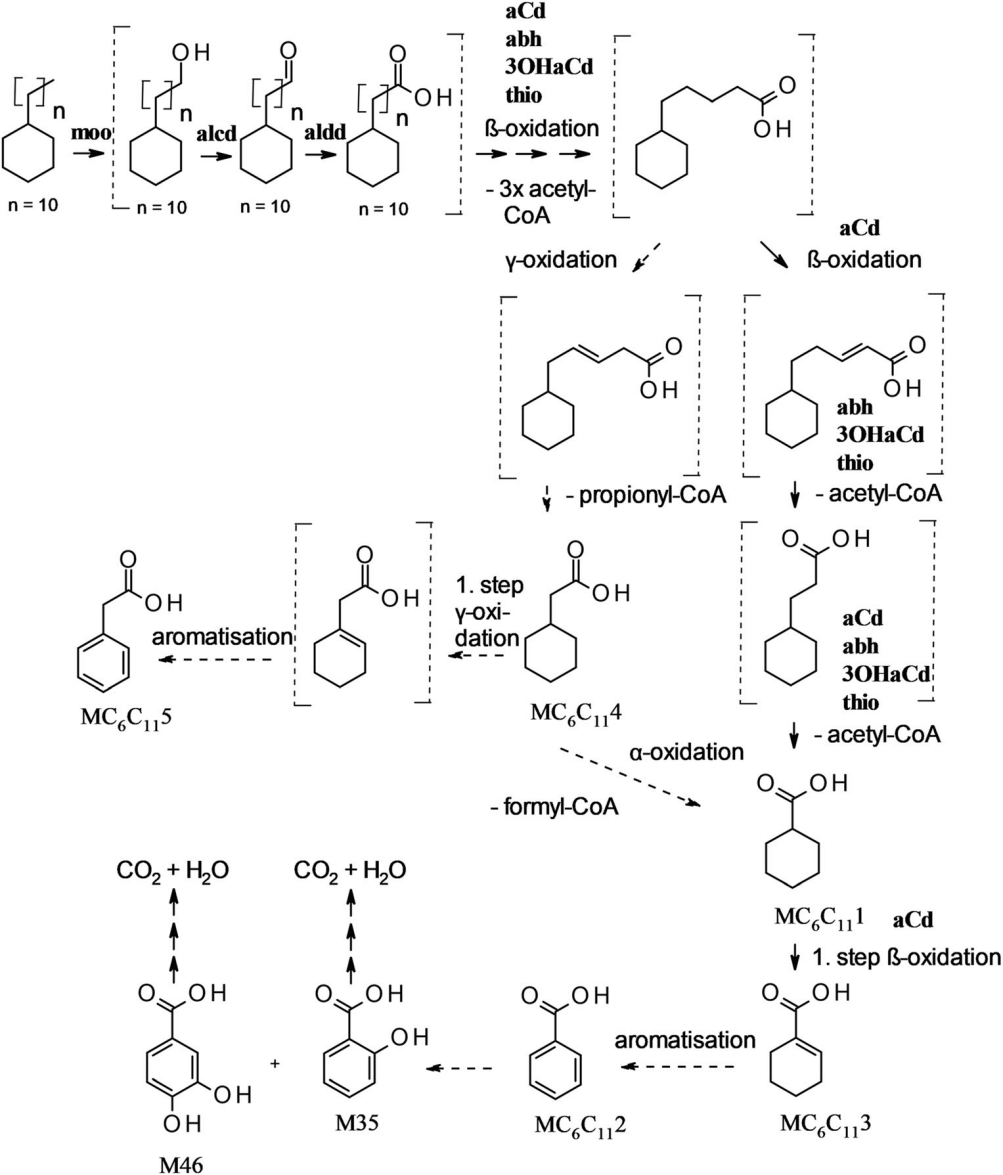
Proposed degradation pathways of *n*-undecylcyclohexane by *M. spathulata* SBUG-Y 2180. MC_6_C_11_1–MC_6_C_11_5 metabolites identified by GC/MS during incubation of *M. spathulata* SBUG-Y 2180 on *n*-undecylcyclohexane. M35 and M46 metabolites identified by GC/MS during growth on crude oil of *M. spathulata* SBUG-Y 2180 (detailed structural characterization of MC_6_C_11_1–MC_6_C_11_5 Supplementary Table S17, of M35 and M46 Supplementary Table S13). Enzymes detected by proteome analyses: moo, monooxygenase; alcd, alcohol dehydrogenase; aldd, aldehyde dehydrogenase; aCd, acyl-CoA dehydrogenase; abh, alpha-beta-hydrolase; 3OHaCd, 3-hydroxyacyl-CoA dehydrogenase; and thio, thiolase. Undetected intermediates in brackets. Solid arrows correspond to proven reaction steps. Dashed arrows correspond to assumed reaction steps

**Fig. 4:**
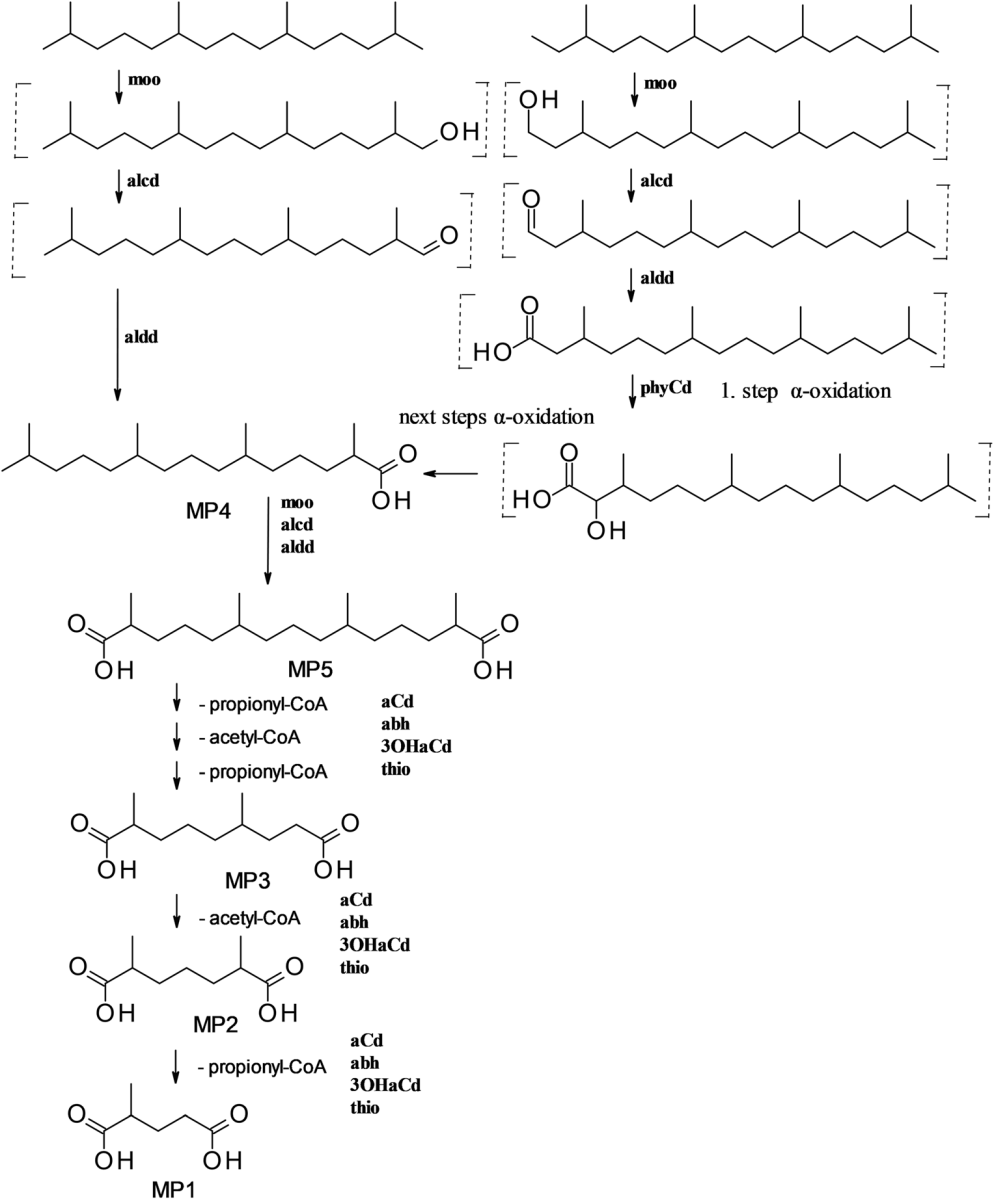
Proposed diterminal oxidation pathway for the degradation of pristane and phytane by *M. spathulata* SBUG-Y 2180 (detailed structural characterization of pristane metabolites in Supplementary Table S15). Enzymes detected by proteome analyses: moo, monooxygenase; alcd, alcohol dehydrogenase; aldd, aldehyde dehydrogenase; aCd, acyl-CoA dehydrogenase; abh, alpha-beta-hydrolase; 3OHaCd, 3-hydroxyacyl-CoA dehydrogenase; thio, thiolase; and phyCd, phytanoyl-CoA dioxygenase. Undetected intermediates in brackets

To study the spectrum of oil components utilized by the strain, we used crude oil with a low content of resins and asphaltenes, on which *M. spathulata* SBUG-Y 2180 grew well. Diesel fuel and fuel oil have a higher content of sulfur, resins, asphaltenes, and other high molecular weight components than crude oil and are consequently more difficult to degrade (Gailiūtė et al. 2011; Khorasani et al. 2013). In line with this, *M. spathulata* SBUG-Y 2180 grew very poorly on medium with fuel oil and poorly on diesel fuel. In addition to growth on crude oil, *M. spathulata* SBUG-Y 2180 can biodegrade an unusually wide spectrum of more than 150 hydrocarbons as substrates (Supplementary Table S8). More than 51 aliphatic and aromatic acids were formed during the degradation of the oil components (Table 2). Thus, this study establishes that yeasts can have oil-degrading properties comparable to those of bacteria (Mikolasch et al. 2015; Mikolasch et al. 2016), which was also described for yeast strains of the genera *Candida* and *Trichosporon* (Farag and Soliman 2011; Gargouri et al. 2015). Proteome analyses of *M. spathulata* showed expression of nearly all potential enzymes necessary for the degradation of alkanes and alkyl chains of cycloaliphatic compounds via initial hydroxylation, transformation to acids, and β-oxidation: monooxygenase (moo), alcohol dehydrogenase (alcd), aldehyde dehydrogenase (aldd), acyl-CoA dehydrogenase (aCd), alpha-beta-hydrolase (abh), 3-hydroxyacyl-CoA dehydrogenase (3OHaCd), and thiolase (thio) (Fig. 3). *M. spathulata* SBUG-Y 2180 is able to transform not only easy-to-degrade *n*-alkanes and *n*-alkyl chains by these enzymes but also polymethyl-substituted alkanes like pristane and phytane (Fig. 4), which are more difficult to deal with. Given the branched-chain alkyl-substituted mono- and dicarboxylic acids MP1 to MP5 (Supplementary Table S15) detected, this yeast used the diterminal degradation pathway as described for bacteria (Mikolasch et al. 2019; Nhi-Cong et al. 2010; Nhi-Cong et al. 2009; Pirnik and McKenna 1977). Phytane is degraded by nearly the same pathway as pristane. The initial steps are also catalyzed by monooxygenase (moo), alcohol dehydrogenase (alcd), and aldehyde dehydrogenase (aldd) forming phytanic acid. Phytanic acid is transformed via α-oxidation. The first enzyme of the α-oxidation, the phytanoyl-CoA dioxygenase (phyCd), was detected by proteome analysis.

Previously, pristane was considered more as an inert carrier for poorly water-soluble substrates during their transformation by yeasts (Green et al. 2000). Alternatively, yeasts have been held to be members of pristane- and phytane-degrading communities of bacteria, yeasts, and fungi (Ururahy et al. 1998), though the roles of the different species in the consumption process were not further investigated.

**Fig. 5:**
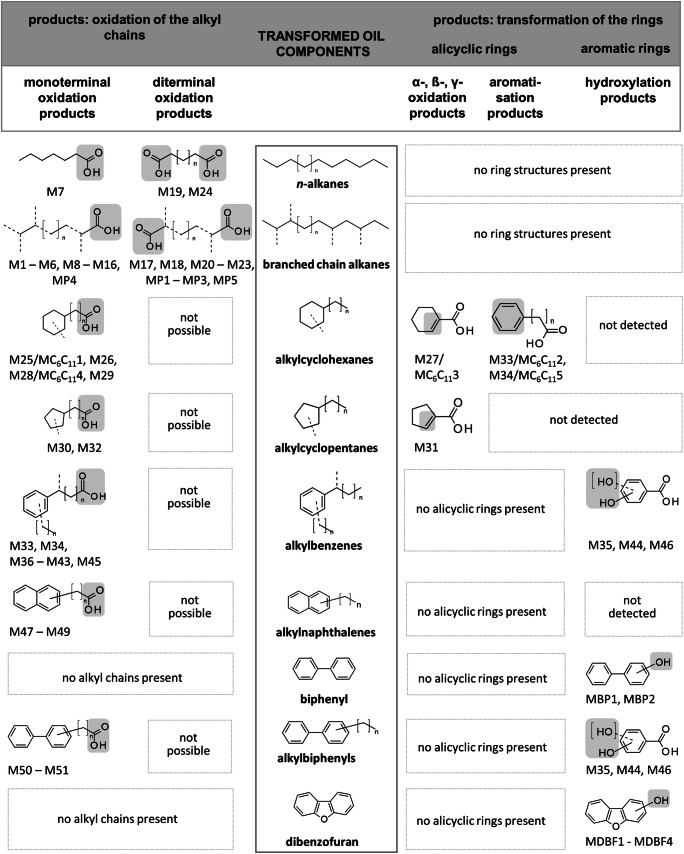
Overview of transformation pathways for the main oil components by *M. spathulata* SBUG-Y 2180

In addition to its degradation of aliphatic hydrocarbons, *M. spathulata* SBUG-Y 2180 is also able to transform aromatic hydrocarbons like biphenyl and dibenzofuran (Table 3). Some enzymes that might be involved in the degradation of aromatic hydrocarbons were found by proteome analyses (Table 4): RSEE01000002.1.g906, RSEE01000002.1.g1287, RSEE01000108.1.g25431, and RSEE01000187.1.g29162. Other yeasts can also transform biphenyls and dibenzofurans. In some cases, ring cleavage products were detected, while in the majority, as with *M. spathulata* SBUG-Y 2180, only hydroxylated products were found (Romero et al. 2002; Schlüter et al. 2013; Sietmann et al. 2006; Sietmann et al. 2000; Zinjarde et al. 2014). However, little is currently known about fungal degradation of alicyclic hydrocarbons, and the alicyclic fraction of oil is among the most degradation-resistant components. Undecylcyclohexane, a representative of the alicyclic fraction of oil, was transformed to five different acids by *M. spathulata* SBUG-Y 2180 (Fig. 3 degradation example *n*-undecylcyclohexane). The first reaction steps are the same as for the degradation of *n*-alkanes—monoterminal oxidation via an alcohol and an aldehyde intermediate to the corresponding carboxylic acid, followed by further β-oxidation (Morgan and Watkinson 1994; Ratledge 1978). A product of these transformations is cyclohexanecarboxylic acid, which was found as an intermediate in our assays and is referred to as MC_6_C_11_1. Furthermore, all enzymes that might possibly catalyze the reactions of this pathway were detected by proteome analyses (Fig. 3). The degradation of the *n*-alkyl chain of *n*-alkylcyclohexanes by additional oxidation mechanisms has been previously proposed (Beam and Perry 1974; Dutta and Harayama 2001; Rontani and Bonin 1992). Both carboxylic acid and acetic acid derivatives were identified from *n*-undecylcyclohexane, *n*-dodecylcyclohexane, *n*-heptadecylcyclohexane, *n*-octadecylcyclohexane, and *n*-nonadecylcyclohexane. The product cyclohexane acetic acid MC_6_C_11_4 was also detected by GC/MS in extracts of *M. spathulata* SBUG-Y 2180 cultivated on *n*-undecylcyclohexane. While Beam and Perry (1974) and Rontani and Bonin (1992) favor α-oxidation as a minor pathway in addition to β-oxidation, Dutta and Harayama (2001) outline γ-oxidation as a further alternative pathway. Regardless of α- or γ-oxidation, we detected both cyclohexanecarboxylic and cyclohexane acetic acid, which indicate pathways parallel to β-oxidation. The γ-pathway does appear to be involved, since the removal of propionyl-CoA from acidic substances has also been described in the degradation of pristane (Fig. 4). Furthermore, we found MC_6_C_11_2, MC_6_C_11_3, and MC_6_C_11_5 as additional transformation products. MC_6_C_11_3 can be an intermediate of further β-oxidation, as shown for the assimilation of cyclohexane acetic acid (Rontani and Bonin 1992). Alternatively, it can be a product of an aromatization process to benzoic acid (Dutta and Harayama 2001), which was also found in our assays, and is referred to as MC_6_C_11_2. The question of how the phenylacetic acid MC_6_C_11_5 is formed must remain open at this point, though we assume a first step of γ-oxidation followed by an aromatization such as for MC_6_C_11_1. On the basis of all the analyzed transformation products, we propose pathways for catabolism of the main oil components by *M. spathulata* SBUG-Y 2180 (Fig. 5).

*M. spathulata* SBUG-Y 2180 is able to transform more than 150 compounds from 9 different groups present as major components of Kazakh crude oil. This basidiomycete yeast is able to degrade *n*-alkyl, branched-chain, aromatic, and polycyclic aromatic structures. Furthermore, the use of *M. spathulata* SBUG-Y 2180 in plant growth experiments resulted in improvements in germination (15%) and in shoot (44%) and root (20%) growth of barley in the presence of crude oil. The P(T<-t) values support the hypothesis of significant growth increase by the inoculation of seeds with *M. spathulata* SBUG-Y 2180 in oil-containing sand. The inoculation of barley with bacteria of the genera *Gordonia* and *Rhodococcus* or the inoculation with culture mixtures of the genera *Rhodococcus* and *Bacillus* or *Rhodococcus* and *Sphingobacterium* promoted the growth of barley in the same way as *M. spathulata* SBUG-Y 2180 (Mikolasch et al. 2015; Mikolasch et al. 2016), in which the effects to the shoot growth are comparable, but the root growth promotion of *M. spathulata* SBUG-Y 2180 is less than by the named bacteria.

In addition to the degradation of the crude oil components, a large number of acidic products were detected during the incubation with crude oil, or with crude oil components such as pristane as a model for polymethyl-substituted alkanes, and undecylcyclohexane as a model for alicyclic compounds. Root exudates containing organic acids, amino acids, and carbohydrates can create a specific microenvironment in the root zone system (Kumar et al. 2006), can change the pH of the environment, and can provide optimal conditions for growth of the rhizosphere microbiota (Gerhardt et al. 2009; Kuiper et al. 2004). In this light, the production of organic acids by *M. spathulata* SBUG-Y 2180 in the soil may also benefit the development of other microorganisms, and thus contribute to the growth increases of barley seen in the inoculation experiments.

In turn, the presence of barley increased the development of the yeast *M. spathulata* SBUG-Y 2180, as seen by the 19% higher number of CFUs in the assay with barley, *M. spathulata* SBUG-Y 2180, and oil as compared to the assay without barley. Thus, *M. spathulata* SBUG-Y 2180 and barley may be powerful partners in the interaction with oil pollutants of soil, and hence for bioremediation of oil-polluted areas. Barley grown on contaminated sites can subsequently be used for the production of bio-gas or bio-fuels (Gatta et al. 2013; Neves et al. 2006; Qureshi et al. 2014; Yang et al. 2015).

## Supplementary Information

ESM 1 (PDF 330 kb)
