# Supplementary material for: Moniliella spathulata, an oil-degrading yeast, which promotes growth of barley in oil-polluted soil
Source: Appl Microbiol Biotechnol. 2020 Nov 20;105(1):401–15. doi: 10.1007/s00253-020-11011-1 (PMC7778628; doi:10.1007/s00253-020-11011-1)
Supplement: Supplementary file 1 — (PDF 330 kb) [file 253_2020_11011_MOESM1_ESM.pdf]

Applied Microbiology and Biotechnology

Supplementary material

Moniliella spathulata an oil-degrading yeast, which promotes growth of barley in oil polluted soil

Annett Mikolasch[1]*, Ramza Berzhanova[2], Anel Omirbekova[2], Anne Reinhard[1], Daniele Zühlke[1], Mareike Meister[3], Togzhan Mukasheva[2], Katharina Riedel[1], Tim Urich[1], Frieder Schauer[1†]

[1] University Greifswald, Institute of Microbiology, Felix-Hausdorff-Straße 8, 17489 Greifswald, Germany
[2] Al-Farabi Kazakh National University, Department of Biology and Biotechnology, Al-Farabi Ave 71, 050040 Almaty, Kazakhstan
[3] Leibniz Institute for Plasma Science and Technology (INP), Felix-Hausdorff-Str. 2, 17489 Greifswald, Germany

* Corresponding author
Phone: ++49-3834-420-5917
Fax: ++49-3834-420-5902
Email: annett.mikolasch@uni-greifswald.de

Supplementary Table S1. Results of identification of the isolated yeast SBUG-Y 2180 by ITS gene sequence analysis of the NCBI nr database.

Isolate	Result of identification	ITS gene sequence analysis [a)]						
		Description	*Max score*	*Total score*	*Query cover*	*E value*	*Ident*	*Accession*
SBUG-Y 2180	*Moniliella spathulata*	Moniliella spathulata strain CBS 241.79 18S ribosomal RNA gene, partial sequence; internal transcribed spacer 1, 5.8S ribosomal RNA gene, and internal transcribed spacer 2, complete sequence; and 28S ribosomal RNA gene, partial sequence	756	756	100%	0	98.38%	KF706432.1
		Trichosporonoides sp. SZ8Y6 internal transcribed spacer 1, partial sequence; 5.8S ribosomal RNA gene and internal transcribed spacer 2, complete sequence; and 28S ribosomal RNA gene, partial sequence	640	640	89%	2.00E-179	96.88%	JF781403.1
		Trichosporonoides sp. SZ8Y3 internal transcribed spacer 1, partial sequence; 5.8S ribosomal RNA gene and internal transcribed spacer 2, complete sequence; and 28S ribosomal RNA gene, partial sequence	636	636	87%	3.00E-178	97.11%	JF781420.1
		Moniliella sp. strain AUMC 10745 internal transcribed spacer 1, partial sequence; 5.8S ribosomal RNA gene and internal transcribed spacer 2, complete sequence; and large subunit ribosomal RNA gene, partial sequence	621	621	91%	7.00E-174	95.00%	KX421105.1
		Trichosporonoides sp. SZ8Y8 internal transcribed spacer 1, partial sequence; 5.8S ribosomal RNA gene and internal transcribed spacer 2, complete sequence; and 28S ribosomal RNA gene, partial sequence	608	608	88%	6.00E-170	95.56%	JF781418.1
		Uncultured eukaryote clone HMPStoolITS2OTU869 5.8S ribosomal RNA gene, partial sequence; internal transcribed spacer 2, complete sequence; and large subunit ribosomal RNA gene, partial sequence	473	473	61%	2.00E-129	98.87%	KY935353.1
		Moniliella dehoogii isolate strain KFP 211T internal transcribed spacer 1, partial sequence; 5.8S ribosomal RNA gene, complete sequence; and internal transcribed spacer 2, partial sequence	331	331	70%	1.00E-86	86.41%	KY946755.1
		Moniliella sp. isolate strain CBS 223.32 internal transcribed spacer 1, partial sequence; 5.8S ribosomal RNA gene, complete sequence; and internal transcribed spacer 2, partial sequence	329	329	73%	5.00E-86	85.76%	KY946753.1

[a)] first eight hits of the NCBI (National Center for Biotechnology Information) BLAST nucleotide collection (nr/nt) are presented

Supplementary Table S2. Results of identification of the isolated yeast SBUG-Y 2180 by ITS gene sequence analysis of mycobank.

Isolate	Result of identification	ITS gene sequence analysis [a)]							
		Reference description	*Score*	*Probability*	*Similarity%*	*Fragments*	*Overlap%*	*Direction*	*Rating*
SBUG-Y 2180	*Moniliella spathulata*	CBS 241.79 Moniliella spathulata, Moniliella spathulata, Type of Trichosporonoides spathulata de Hoog, India, nlink4056: publicly available rDNA ITS sequences FunCBS	586.436	4.64E-167	99.74	1	89.07	+/-	* * *
		KF706432 Fungi, Basidiomycota, Moniliellomycetes, Moniliellales, Moniliellaceae, Moniliella, Moniliella spathulata UNITE	629.23	1.16E-178	97.448	1	100	+/-	* * *
		JF781420 Fungi, Basidiomycota, Moniliellomycetes, Moniliellales, Moniliellaceae, Trichosporonoides, Trichosporonoides sp UNITE	500.848	5.12E-140	96.842	1	87.907	+/-	* * *
		JF781403 Fungi, Basidiomycota, Moniliellomycetes, Moniliellales, Moniliellaceae, Trichosporonoides, Trichosporonoides sp	505.603	1.90E-141	96.623	1	89.07	+/-	* * *
		KY935353 Protista, unidentified, unidentified, unidentified, unidentified, unidentified, Protista sp UNITE	393.071	1.43E-107	98.872	1	61.628	+/-	* *
		JF781419 Fungi, Basidiomycota, Moniliellomycetes, Moniliellales, Moniliellaceae, Trichosporonoides, Trichosporonoides sp UNITE	472.319	1.99E-131	92.388	1	88.14	+/-	* *
		JF781418 Fungi, Basidiomycota, Moniliellomycetes, Moniliellales, Moniliellaceae, Trichosporonoides, Trichosporonoides sp UNITE	472.319	1.99E-131	92.388	1	88.14	+/-	* *
		KX421105 Fungi, Basidiomycota, Moniliellomycetes, Moniliellales, Moniliellaceae, Moniliella, Moniliella sp UNITE	472.319	1.99E-131	92.388	1	88.14	+/-	* *
		SH1244131.08FU EU252153 Fungi, Basidiomycota, Moniliellomycetes, Moniliellales, Moniliellaceae, Moniliella, Moniliella sp UNITE	226.65	1.78E-57	95	3	55.814	+/-	*
		CBS 126564 Moniliella, Moniliella, Type of Moniliella dehoogii, Vietnam, nlink4056: publicly available rDNA ITS sequences	350.277	5.72E-96	85.846	1	75.349	+/-	*

[a)] first ten hits of the mycobank analysis are presented

Supplementary Table S3. Results of identification of the purchased yeast material *Moniliella spathulata* strain CBS 241.79 by ITS gene sequence analysis of the NCBI nr database.

Bought yeast material	Result of identification	ITS gene sequence analysis [a)]						
		Description	*Max score*	*Total score*	*Query cover*	*E value*	*Ident*	*Accession*
Moniliella spathulata strain CBS 241.79	*Moniliella spathulata*	Moniliella spathulata strain CBS 241.79 18S ribosomal RNA gene, partial sequence; internal transcribed spacer 1, 5.8S ribosomal RNA gene, and internal transcribed spacer 2, complete sequence; and 28S ribosomal RNA gene, partial sequence	527	527	99%	1.00E-145	99.65%	KF706432.1
		Trichosporonoides sp. SZ8Y6 internal transcribed spacer 1, partial sequence; 5.8S ribosomal RNA gene and internal transcribed spacer 2, complete sequence; and 28S ribosomal RNA gene, partial sequence	477	477	94%	1.00E-130	98.18%	JF781403.1
		Trichosporonoides sp. SZ8Y3 internal transcribed spacer 1, partial sequence; 5.8S ribosomal RNA gene and internal transcribed spacer 2, complete sequence; and 28S ribosomal RNA gene, partial sequence	473	473	93%	1.00E-129	98.52%	JF781420.1
		Moniliella sp. strain AUMC 10745 internal transcribed spacer 1, partial sequence; 5.8S ribosomal RNA gene and internal transcribed spacer 2, complete sequence; and large subunit ribosomal RNA gene, partial sequence	457	457	98%	1.00E-124	95.52%	KX421105.1
		Trichosporonoides sp. SZ8Y8 internal transcribed spacer 1, partial sequence; 5.8S ribosomal RNA gene and internal transcribed spacer 2, complete sequence; and 28S ribosomal RNA gene, partial sequence	446	446	93%	3.00E-121	96.34%	JF781418.1
		Moniliella carnis isolate strain KFP 246T internal transcribed spacer 1, partial sequence; 5.8S ribosomal RNA gene, complete sequence; and internal transcribed spacer 2, partial sequence	313	313	71%	3.00E-81	94.17%	KY946756.1
		Moniliella dehoogii isolate strain KFP 211T internal transcribed spacer 1, partial sequence; 5.8S ribosomal RNA gene, complete sequence; and internal transcribed spacer 2, partial sequence	313	313	69%	3.00E-81	94.61%	KY946755.1
		Uncultured eukaryote clone HMPStoolITS2OTU869 5.8S ribosomal RNA gene, partial sequence; internal transcribed spacer 2, complete sequence; and large subunit ribosomal RNA gene, partial sequence	291	291	54%	2.00E-74	100.0%	KY935353.1

[a)] first eight hits of the NCBI (National Center for Biotechnology Information) BLAST nucleotide collection (nr/nt) are presented

Supplementary Table S4. Results of identification of the purchased yeast material *Moniliella spathulata* strain CBS 241.79 by ITS gene sequence analysis of mycobank.

Bought yeast material	Result of identification	ITS gene sequence analysis [a]							
		Reference description	*Score*	*Probability*	*Similarity%*	*Fragments*	*Overlap%*	*Direction*	*Rating*
Moniliella spathulata strain CBS 241.79	*Moniliella spathulata*	CBS 241.79 Moniliella spathulata, Moniliella spathulata, Type of Trichosporonoides spathulata de Hoog, India, nlink4056: publicly available rDNA ITS sequences FunCBS	491.338	2.79E-138	94.017	1	58.893	+/+	*
		KF706432 Fungi, Basidiomycota, Moniliellomycetes, Moniliellales, Moniliellaceae, Moniliella, Moniliella spathulata UNITE	534.132	7.01E-150	89.336	1	70.805	+/+	*
		JF781420 Fungi, Basidiomycota, Moniliellomycetes, Moniliellales, Moniliellaceae, Trichosporonoides, Trichosporonoides sp UNITE	437.45	8.91E-121	89.147	1	64.765	+/+	*
		JF781403 Fungi, Basidiomycota, Moniliellomycetes, Moniliellales, Moniliellaceae, Trichosporonoides, Trichosporonoides sp UNITE	443.79	1.10E-122	88.665	1	66.443	+/+	*
		JF781418 Fungi, Basidiomycota, Moniliellomycetes, Moniliellales, Moniliellaceae, Trichosporonoides, Trichosporonoides sp UNITE	408.92	3.45E-112	84.794	1	64.933	+/+	*
		JF781419 Fungi, Basidiomycota, Moniliellomycetes, Moniliellales, Moniliellaceae, Trichosporonoides, Trichosporonoides sp UNITE	408.92	3.45E-112	84.794	1	64.933	+/+	*
		KX421105 Fungi, Basidiomycota, Moniliellomycetes, Moniliellales, Moniliellaceae, Moniliella, Moniliella sp UNITE	413.675	1.28E-113	83.871	1	67.45	+/+	*

[a] the seven hits of the mycobank analysis are presented

Supplementary Table S5. Result of ITS1 sequence alignment of the isolated yeast *M. spathulata* SBUG-Y 2180 and the purchased cell material of the *Moniliella spathulata* strain CBS 241.79 using the blastn algorithm.

**ITS gene sequence alignment**						
Description	*Max score*	*Total score*	*Query cover*	*E value*	*Ident*	*Accession*
SBUG-Y 2180_ITS1 -- 16..443 of sequence against *Moniliella spathulata* Cb_ITS1 -- 14.303 of sequence	534	534	99%	1.00E-156	100.00%	Query_45897

Supplementary Table S6. Result of assembled ITS sequence alignment of the isolated yeast *M. spathulata* SBUG-Y 2180 and the purchased cell material of the *Moniliella spathulata* strain CBS 241.79 using the blastn algorithm.

**ITS gene sequence alignment**						
Description	*Max score*	*Total score*	*Query cover*	*E value*	*Ident*	*Accession*
SBUG-Y 2180 ITS_alignment -- against *Moniliella spathulata* Cb_ITS_alignment	527	527	99%	2.00E-154	99.65%	Query_48627

Supplementary Table S7. Results of the 32 miniaturized assimilation tests within the API 32 C test strip of the isolated yeast *M. spathulata* SBUG-Y 2180 and the purchased cell material of the *Moniliella spathulata* strain CBS 241.79 and literature data of *Moniliella spathulata*

**Test**	**Substrate**	***M. spathulata* SBUG-Y 2180**	***Moniliella spathulata* strain CBS 241.79**	***Moniliella spathulata described by Kurtzman et al. (2011)***
GAL	D-Galactose	-	-	v
ACT	Cycloheximide (actidione)	-	-	n
SAC	D-Saccharose (sucrose)	+	+	+
NAG	N-Acetyl-glucosamine	+	+	n
LAT	Lactic acid	-	-	+
ARA	L-Arabinose	-	-	-
CEL	D-Cellobiose	+	+	+
RAF	D-Raffinose	+	+	+
MAL	D-Maltose	-	-	+
TRE	D-Trehalose	-	-	-
2KG	Potassium 2-ketogluconate	-	-	n
MDG	Methyl α-D-glucopyranoside	-	-	-
MAN	D-Mannitol	+	+	+
LAC	D-Lactose	-	-	v
INO	Inositol	-	-	-
0	No substrate	-	-	-
SOR	D-Sorbitol (D-glucitol)	+	+	+
XYL	D-Xylose	-	-	v
RIB	D-Ribose	+	+	+
GLY	Glycerol	+	+	+
RHA	L-Rhamnose	-	-	-
PLE	Palatinose	-	-	n
ERY	Erythritol	+	+	+
MEL	D-Melibiose	-	-	-
GRT	Sodium glucuronate	-	-	n
MLZ	D-Melezitose	-	-	+
GNT	Potassium gluconate	+	+	n
LVT	Levulinic acid (levulinate)	-	-	n
GLU	D-Glucose	+	+	+
SBE	L-Sorbose	-	-	-
GLN	Glucosamine	-	-	n
ESC	Esculin ferric citrate	+	+	n

+ positive
no data
V variable
- negative
gray table rows indicate different test results for *M. spathulata* SBUG-Y 2180, the purchased cell material of the *Moniliella spathulata* strain CBS 241.79 and literature data of *Moniliella spathulata*

Supplementary Table S8. **Aliphatic and aromatic components of crude oil detected by GC/MS and after growth of *M. spathulata* SBUG-Y 2180 on crude oil.**

**Detected oil components**			**Transformation of oil components** [a)]			
Name	*Structure*	*Rf [min]*	*Number of transformed components of the substance group*	*Amount of transformation [%]*[b)]		
				7d	*14d*	*28d*
***n*-alkanes** (23 detected compounds)						
decane to dotriacontane	$C_{10}H_{22}$ to $C_{32}H_{66}$	6.2 to 32.5	23	100 [c)]	100 [c)]	100 [c)]
**alkylcyclohexanes**						
n-alkylcyclohexanes (23 detected compounds)						
ethylcyclohexane to octadecylcyclohexane	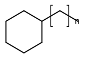 n= 2 - 24	5.0 to 33.4	23	31 to 61	18 to 100 [c)]	32 to 100 [c)]
n-alkyl-methylcyclohexanes (40 detected compounds)						
methyl-substituted *n*-alkylcyclohexanes	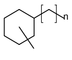 n= 1 - 21	4.3 to 31.5	40	55 to 100 [c)]	100 [c)]	100 [c)]
**alkylcyclopentanes**						
n-alkylcyclopentanes (compounds detected in traces)						
n-alkylcyclopentanes	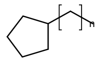 n= 5 - x	8.4 to 20.0	6	100 [c)]	100 [c)]	100 [c)]
n-alkyl-methylcyclopentanes (compounds detected in traces)						
methyl-substituted *n*-alkylcyclopentanes	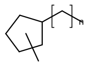	8.5 to 20.5	4	100 [c)]	100 [c)]	100 [c)]
**branched chain alkanes**						
monomethyl- and monoethyl-substituted alkanes (19 detected compounds)						
monomethyl- and monoethyl-substituted alkanes	C_9H_{20} to $C_{23}H_{48}$	6.1 to 25.0	15	100 [c)]	100 [c)]	100 [c)]
polymethyl-substituted alkanes						
2,6,10-trimethyldodecane	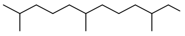	13.0	1	24	18	34
2,6,10-trimethylpentadecane	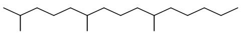	17.2	1	38	38	39
pristane (2,6,10,14-tetramethylpentadecane)	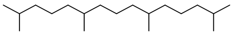	18.0	1	24	22	23
2,6,10,14-tetramethylhexadecane	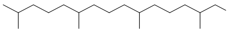	19.4	1	23	23	20
**alkylbenzenes**						
n-alkyl-substituted benzenes	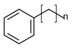 n = 3-20	7.3 to 30.2	16	16 to 100 [c)]	37 to 100 [c)]	42 to 100 [c)]
poly-*n*-alkyl-substituted benzenes (seven well-detected and traces of longer compounds)	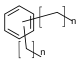 n = 0-x	4.7 to 20.0	7	100 [c)]	100 [c)]	100 [c)]
branched-chain-alkyl-substituted benzenes (three well-detected and traces of longer compounds)	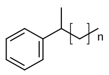 n = 0-x	5.6 to 20.0	3	100 [c)]	100 [c)]	100 [c)]
**naphthalenes**						
naphthalene	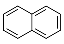	10.5	1	0	31	100 [c)]
n-alkyl-substituted naphthalenes (four well-detected and traces of longer alkylnaphthalenes)	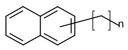 n = 0-x	12.4 to 30.0	4	0	5 to 19	20 to 65
**biphenyls**						
biphenyl	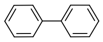	13.8	1	10	24	49
n-alkyl-substituted biphenyls (three well-detected and traces of longer alkylbiphenyls)	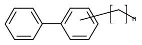 n = 0-x	15.5 to 30.0	3	0	0 to 15	7 to 22

[a)] not all detected oil components are listed, only the transformation of well-detected substances which could be analyzed in extracts after alkaline extraction (pH9) of MSMD and crude oil (1 mL in 100 mL MSMD) are shown [b)] The peak areas of the control assays with only oil in medium were set to 100 % and the peak areas of the transformation assays were calculated as x % of the peak areas of the controls. Then we express transformation as 100 – x % = y%. [c)] not detectable after incubation

Supplementary Table S9. Mass spectrometric data of *n*-alkyl- and branched chain alkyl-substituted monocarboxylic acids identified during growth on crude oil of *M. spathulata* SBUG-Y 2180. These compounds were analysed by GC/MS. The extracted acids were transformed for analytical purposes by methylation to the corresponding methyl esters.

No	t_R (min)	Metabolites	m/z (%) (70 eV) - analyzed with GC/MS
M1	5.1<br>5.4 st	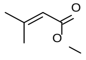	39 (16.0), 53 (10.0), 55 (36.0), 67 (2.0), 82 (13.0), 83 (100.0), 99 (6.0), 114 (43.0)<br>Standard: 39 (17.0), 53 (10.0), 55 (38.0), 67 (2.0), 82 (14.0), 83 (100.0), 99 (6.0), 114 (46.0)
M2	5.2	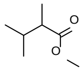	41 (13.0), 43 (26.0), 55 (10.0), 56 (9.0), 57 (26.0), 59 (20.0), 71 (20.0), 83 (3.0), 88 (100.0), 99 (11.0), 115 (10.0)<br>41, 43, 55, 56, 57, 59, 71, 83, 88, 99, 115 standard from the library NIST
M3	5.8	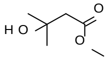	43 (100.0), 59 (98.0), 73 (8.0), 74 (25.0), 75 (10.0), 85 (59.0), 101 (7.0), 117 (51.0)<br>43, 59, 73, 74, 75, 85, 101, 117 standard from the library NIST
M4	6.1<br>6.4 st	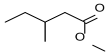	41 (16.0), 43 (27.0), 59 (23.0), 74 (100.0), 87 (3.0), 99 (17.0), 101 (14.0), 115 (2.0)<br>Standard 41 (16.0), 43 (29.0), 59 (23.0), 74 (100.0), 87 (3.0), 99 (17.0), 101 (14.0), 115 (1.0),
M5	8.0	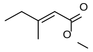	39 (24.0), 41 (59.0), 53 (14.0), 55 (9.0), 59 (13.0), 67 (22.0), 68 (13.0), 69 (25.0), 81 (15.0), 95 (15.0), 96 (25.0), 97 (100.0), 113 (15.0), 128 (62.0)<br>39, 41, 53, 55, 59, 67, 68, 69, 81, 95, 96, 97, 113, 128 standard from the library NIST
M6	8.6	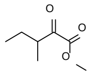	39 (7.0), 41 (34.0), 55 (6.0), 56 (7.0), 57 (100.0), 58 (4.0), 59 (7.0), 69 (3.0), 85 (39.0), 101 (1.0), 144 (2.0)<br>39, 41, 55, 56, 57, 58, 59, 69, 85, 101, 144 standard from the library NIST
M7	8.7<br>8.9 st	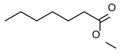	43 (31.0), 74 (100.0), 87 (6.0), 101 (17.0), 113 (12.0), 144 (1.0)<br>Standard: 43 (27.0), 74 (100.0), 87 (36.0), 101 (15.0), 113 (16.0), 144 (1.0)
M8	9.6	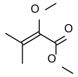	39 (32.0), 41 (32.0), 43 (100.0), 45 (45.0), 53 (15.0), 55 (71.0), 59 (28.0), 69 (13.0), 71 (54.0), 73 (24.0), 83 (37.0), 85 (53.0), 99 (9.0), 100 (8.0), 101 (7.0), 111 (6.0), 129 (73.0), 143 (1.0)<br>39, 41, 43, 45, 53, 55, 59, 69, 71, 73, 83, 85, 99, 100, 101, 111, 129, 143 standard from the library NIST
M9	10.9	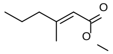	39 (37.0), 41 (38.0), 53 (23.0), 54 (10.0), 55 (70.0), 59 (28.0), 67 (37.0), 69 (55.0), 82 (55.0), 83 (45.0), 85 (18.0), 95 (63.0), 111 (100.0), 114 (58.0), 127 (53.0), 142 (47.0)<br>39, 41, 53, 54, 55, 59, 67, 69, 82, 83, 85, 95, 111, 114, 127, 142 standard from the library NIST
M10	11.2	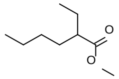	41 (26.0), 43 (15.0), 55 (21.0), 57 (39.0), 59 (13.0), 69 (9.0), 87 (100.0), 101 (20.0), 102 (87.0), 115 (13.0), 130 (14.0)<br>41, 43, 55, 57, 59, 69, 87, 101, 102, 115, 130 standard from the library NIST
M11	12.2	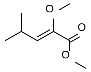	41(34.0), 43 (69.0), 45 (46.0), 55 (25.0), 57 (26.0), 59 (24.0), 69 (11.0), 73 (10.0), 79 (9.0), 81 (12.0), 83 (19.0), 85 (24.0), 99 (8.0), 100 (5.0), 108 (6.0), 115 (100.0), 125 (14.0), 158 (1.0)<br>41, 43, 45, 55, 57, 59, 69, 73, 79, 81, 83, 85, 99, 100, 108, 115, 125, 158 standard from the library NIST

Continuation Supplementary Table S9

No	t_R (min)	Metabolites	m/z (%) (70 eV) - analyzed with GC/MS
M12	14.1	with monomethyl branching point 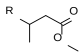	41 (19.0), 43 (23.0), 55 (18.0), 57 (17.0), 59 (13.0), 69 (15.0), 73 (6.0), 74 (100.0), 75. (13.0), 83 (10.0), 101. (51.0), 141 (5.0)<br>structure deduced from fragmentation and retention time
M13	17.5	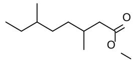	41 (20.0), 43 (25.0), 55. (17.0), 57 (10.0), 59 (13.0), 69 (20.0), 73 (6.0), 74 (100.0), 75 (16.0), 83 (6.0), 101 (68.0), 129 (3.0), 155 (4.0), 171 (2.0), 186 (1.0)<br>41, 43, 55, 57, 59, 69, 73, 74, 75, 83, 101, 129, 155, 171 standard from the library NIST
M14	17.7	isomeric compound of M13	41 (24.0), 43 (19.0), 55 (29.0), 57 (15.0), 59 (15.0), 69 (20.0), 73 (7.0), 74 (100.0), 75 (14.0), 83 (17.0), 101 (78.0), 129 (9.0), 155 (5.0), 171 (2.0), 186 (1.0)<br>41, 43, 55, 57, 59, 69, 73, 74, 75, 83, 101, 129, 155, 171 standard from the library NIST of M13
M15	18.6	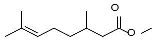	39 (17.0), 41 (62.0), 53 (11.0), 55 (32.0), 56 (9.0), 59 (21.0), 67 (21.0), 68 (16.0), 69 (100.0), 70 (15.0), 73 (17.0), 74 (17.0), 81 (13.0), 82 (30.0), 83 (9.0), 94 (24.0), 95 (67.0), 96 (14.0), 101 (10.0), 109 (33.0), 110 (63.0), 119 (3.0), 129 (7.0), 137 (4.0), 152 (40.0), 153 (15.0), 184 (7.0)<br>39, 41, 55, 53, 56, 59, 67, 68, 69, 70, 73, 74, 81, 82, 83, 94, 95, 96, 101, 109, 110, 119, 129, 137, 152, 153, 184 standard from the library NIST
M16	18.8	isomeric compound of M13	41 (15.0), 43 (19.0), 55 (12.0), 57 (6.0), 59 (11), 69 (12.0), 73 (5.0), 74 (100.0), 75 (16.0), 83 (5.0), 101 (42.0), 155 (7.0), 171 (1.0), 186 (1.0)<br>41, 43, 55, 57, 59, 69, 73, 74, 75, 83, 101, 155, 171 standard from the library NIST of M13

(st: purchased standard; standard from the library NIST only shows the fragment without the intensity)

Supplementary Table S10. Mass spectrometric data of *n*-alkyl- and branched chain alkyl-substituted dicarboxylic acids identified during growth on crude oil of *M. spathulata* SBUG-Y 2180. These compounds were analysed by GC/MS. The extracted acids were transformed for analytical purposes by methylation to the corresponding methyl esters.

No	t_R (min)	Metabolites	m/z (%) (70 eV) - analyzed with GC/MS
M17	13.5<br>13.7 st	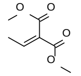	39 (27.0), 59 (43.0), 68 (32.0), 98 (27.0), 126 (100.0), 127 (46.0), 158 (1.0)<br>Standard: 39 (16.0), 59 (44.0), 68 (39.0), 98 (25.0), 126 (100.0), 127 (80.0), 158 (2.0)
M18	15.5<br>15.5 st	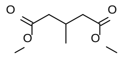	39 (21.0), 41 (35.0), 42 (25.0), 43 (33.0), 55 (43.0), 59 (99.0), 69 (75.0), 71 (17.0), 73 (54.0), 74 (37.0), 82 (31.0), 83 (20.0), 101 (88.0), 114 (100.0), 115 (43.0), 142 (32.0), 143 (81.0)<br>Standard: 39 (19.0), 41 (31.0), 42 (26.0), 43 (29.0), 55 (39.0), 59 (96.0), 69 (70.0), 71 (12.0), 73 (54.0), 74 (32.0), 82 (28.0), 83 (18.0), 101 (83.0), 114 (100.0), 115 (42.0), 142 (30.0), 143 (81.0)
M19	18.0<br>18.1 st	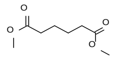	39 (12.0), 41(19.0), 43 (24.0), 55 (79.0), 59 (100.0), 73 (33.0), 74 (39.0), 82 (10.0), 83 (29.0), 101 (71.0), 111 (74.0), 114 (90.0), 115 (17.0), 142 (15.0), 143 (58.0)<br>Standard: 39 (11.0), 41 (22.0), 43 (24.0), 55 (80.0), 59 (100.0), 73 (32.0), 74 (44.0), 82 (12.0), 83 (28.0), 101 (73.0), 111 (75.0), 114 (98.0), 115 (21.0), 142 (15.0), 143 (61.0)
M20	19.2	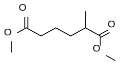	39 (12.0), 41 (49.0), 42 (24.0), 43 (33.0), 55 (42.0), 56 (18.0), 57 (39.0), 59 (84.0), 68 (25.0), 69 (100.0), 74 (40.0), 87 (34.0), 88 (100.0), 97 (47.0), 101 (22.0), 109 (1.0), 113 (19.0), 128 (74.0), 129 (49.0), 157 (30.0)<br>39, 41, 42, 43, 55, 56, 57, 59, 68, 69, 74, 87, 88, 97, 101, 109, 113, 128, 129, 157 standard from the library NIST
M21	19.5<br>19.5 st	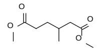	39 (13.0), 41 (31.0), 43 (25.0), 55 (64.0), 59 (59.0), 69 (66,0), 73 (68.0), 74 (47,0), 83 (38.0), 87 (25.0), 96 (20.0), 97 (19.0), 101 (11.0), 114 (14.0), 115 (100.0), 125 (57.0), 128 (56.0), 141 (2.0), 157 (41.0), 188 (1.0)<br>Standard: 39 (18.0), 41 (39.0), 43 (29.0), 55 (74.0), 59 (69.0), 69 (76.0), 73 (72.0), 74 (47.0), 83 (42.0), 87 (27.0), 96 (22.0), 97 (20.0), 101 (12.0), 114 (14.0), 115 (100.0), 125 (59.0), 128 (57.0), 141 (2.0), 157 (40.0), 188 (1.0)
M22	22.7	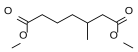	41 (34.0), 42 (16.0), 43 (19.0), 55 (41.0), 59 (48.0), 69 (100.0), 74 (56.0), 82 (26.0), 83 (25.0), 87 (31.0), 97 (74.0), 101 (17.0), 110 (11.0), 111 (17.0), 125 (14.0), 127 (10.0), 128 (13.0), 129 (98.0), 138 (11.0), 139 (26.0), 142 (24.0), 155 (6.0), 171 (29.0)<br>41, 42, 43, 55, 59, 69, 74, 82, 83, 87, 97, 101, 110, 111, 125, 127, 128, 129, 138, 139, 142, 155, 171 standard from the library NIST
M23	22.9	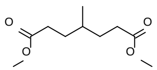	41 (40.0), 55 (44.0), 69 (100.0), 83 (23.0), 97 (67.0), 111 (14.0), 129 (77.0), 142 (17.0), 171 (21.0)<br>41, 55, 69, 83, 97, 111, 129, 142, 171 standard from the library NIST
M24	28.8	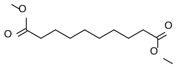	39 (13.0), 41 (44.0), 42 (16.0), 43 (44.0), 55 (100.0), 56 (12.0), 59 (63.0), 67 (14.0), 69 (60.0), 73 (15.0), 74 (87.0), 81 (21.0), 82 (11.0), 83 (53.0), 84 (22.0), 87 (30.0), 95 (13.0), 96 (20.0), 97 (78.0), 98 (34.0), 101 (32.0), 124 (20.0), 125 (82.0), 138 (31.0), 139 (21.0), 157 (58.0), 166 (50.0), 199 (45.0)<br>39, 41, 42, 43, 55, 56, 59, 67, 69, 73, 74, 81, 82, 83, 84, 87, 95, 96, 97, 98, 101, 124, 125, 138, 139, 157, 166, 199 standard from the library NIST

(st: purchased standard; standard from the library NIST only shows the fragment without the intensity)

Supplementary Table S11. Mass spectrometric data of cyclohexylalkanoic acids identified during growth on crude oil of *M. spathulata* SBUG-Y 2180. These compounds were analysed by GC/MS. The extracted acids were transformed for analytical purposes by methylation to the corresponding methyl esters.

No	t_R (min)	Metabolites	m/z (%) (70 eV) - analyzed with GC/MS
M25	11.6<br>11.6 st	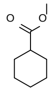	41 (37.0), 55 (100.0), 67 (22.0), 68 (19.0), 69 (13.0), 74 (38.0), 81 (17.0), 82 (27.0), 83 (79.0), 87 (95.0), 101 (15.0), 110 (28.0), 111 (23.0), 113 (23.0), 127 (9.0), 142 (36.0)<br>Standard: 41 (32.0), 55 (92.0), 67 (21.0), 68 (19.0), 69 (13.0), 74 (39.0), 81 (17.0), 82 (28.0), 83 (81.0), 87 (100.0), 101 (17.0), 110 (31.0), 111 (25.0), 113 (26.0), 127 (10.0), 142 (40)
M26	13.8<br>13.9 st	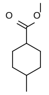	39 (21.0), 41 (23.0), 55 (100.0), 67 (16.0), 70 (26.0), 81 (34.0), 82 (30.0), 87 (38.0), 95 (12.0), 96 (16.0), 97 (52.0), 113 (12.0), 124 (19.0), 125 (30.0), 141 (6.0), 156 (27.0)<br>Standard: 39 (17.0), 41 (24.0), 55 (100.0), 67 (15.0), 70 (26.0), 81 (37.0), 82 (26.0), 87 (40.0), 95 (13.0), 96 (16.0), 97 (53.0), 113 (11.0), 124 (19.0), 125 (14.0), 141 (8.0), 156 (27.0)
M27	14.3<br>14.3 st	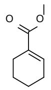	39 (10.0), 41 (10.0), 51 (11.0), 52 (9.0), 53 (18.0), 65 (4.0), 77 (15.0), 79 (58.0), 80 (57.0), 81 (100.0), 108 (20.0), 109 (30.0), 140 (25.0)<br>Standard: 39 (14.0), 41 (14.0), 51 (10.0), 52 (7.0), 53 (25.0), 65 (6.0), 77 (19.0), 79 (66.0), 80 (71.0), 81 (100.0), 108 (25.0), 109 (26.0), 140 (35.0)
M28	14.8<br>14.8 st	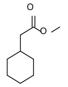	41 (11.0), 43 (17.0), 55 (18.0), 67 (7.0), 74 (100.0), 75 (34.0), 81 (6.0), 97 (5.0), 113 (2.0), 125 (8.0), 156 (1.0)<br>Standard: 41 (9.0), 43 (15.0), 55 (16.0), 67 (7.0), 74 (100.0), 75 (34.0), 81 (6.0), 97 (6.0), 113 (2.0), 125 (9.0), 156 (1.0)
M29	16.9	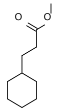	43 (15.0), 55 (17.0), 67 (6.0), 74 (100.0), 75 (34.0), 81 (9.0), 97 (8.0), 170 (1.0)<br>43, 55, 67, 74, 75, 81, 97, 170 standard from the library NIST

(st: purchased standard; standard from the library NIST only shows the fragment without the intensity)

Supplementary Table S12. Mass spectrometric data of cyclopentylalkanoic acids identified during growth on crude oil of *M. spathulata* SBUG-Y 2180. These compounds were analysed by GC/MS. The extracted acids were transformed for analytical purposes by methylation to the corresponding methyl esters.

No	t_R (min)	Metabolites	m/z (%) (70 eV) - analyzed with GC/MS
M30	8.3<br>8.6 st	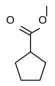	39 (15.0), 41 (33.0), 55 (16.0), 59 (6.0), 67 (13.0), 68 (10.0), 69 (43.0), 74 (3.0), 87 (100.0), 97 (15.0), 100 (19.0), 128 (6.0)<br>Standard: 39 (11.0), 41 (25.0), 55 (12.0), 59 (5.0), 67 (12.0), 68 (10.0), 69 (42.0), 74 (2.0), 87 (100.0), 97 (16.0), 100 (18.0), 128 (8.0),
M31	10.0<br>10.1 st	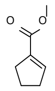	39 (28.0), 41 (33.0), 59 (18.0), 65 (32.0), 66 (28.0), 67 (100.0), 95 (71.0), 111 (8.0), 126 (45.0)<br>Standard: 39 (22.0), 41 (20.0), 59 (8.0), 65 (24.0), 66 (27.0), 67 (100.0), 95 (63.0), 111 (9.0), 126 (47.0),
M32	10.1	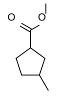	39 (11.0), 41 (19.0), 55 (38.0), 59 (8.0), 67 (15.0), 68 (3.0), 69 (6.0), 83 (23.0), 87 (100.0), 100 (20.0), 111 (12.0), 127 (10.0), 142 (6.0)<br>39, 41, 55, 59, 67, 68, 69, 83, 87, 100, 111, 127, 142 standard from the library NIST

(st: purchased standard; standard from the library NIST only shows the fragment without the intensity)

Supplementary Table S13. Mass spectrometric data of phenylalkanoic acids identified during growth on crude oil of *M. spathulata* SBUG-Y 2180. These compounds were analysed by GC/MS. The extracted acids were transformed for analytical purposes by methylation to the corresponding methyl esters.

No	t_R (min)	Metabolites	m/z (%) (70 eV) - analyzed with GC/MS
M33	12.8<br>12.9 st	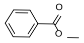	50 (9.0), 51 (20.0), 77 (58.0), 91 (2.0), 92 (2.0), 105 (100.0), 106 (8.0), 136 (35.0)<br>Standard: 50 (7.0), 51 (17.0), 77 (56.0), 91 (2.0), 92 (2.0), 105 (100.0), 106 (8.0), 136 (38.0)
M34	15.7<br>15.8 st	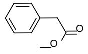	39 (5.0), 51 (3.0), 59 (5.0), 65 (13.0), 77 (1.0), 89 (5.0), 91 (100.0), 92 (8.0), 105 (1.0), 119 (2.0), 150 (30.0)<br>Standard: 39 (4.0), 51 (2.0), 59 (5.0), 65 (12.0), 77 (1.0), 89 (5.0), 91 (100.0), 92 (8.0), 105 (1.0), 119 (2.0), 150 (34.0)
M35	16.3<br>16.3 st	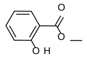	39 (13), 63 (12), 64 (12), 65 (20), 92 (57), 93 (16), 120 (100), 121 (32), 152 (44)<br>Standard: 39 (12), 63 (12), 64 (12), 65 (19), 92 (59), 93 (13), 120 (100), 121 (29), 152 (45)
M36	16.6<br>16.7 st	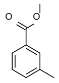	39 (5.0), 50 (2.0), 51 (3.0), 63 (7.0), 65 (17.0), 77 (1.0), 89 (8.0), 90 (4.0), 91 (53.0), 105 (2.0), 119 (100.0), 120 (8.0), 135 (1.0), 150 (36.0)<br>Standard: 39 (4.0), 50 (2.0), 51 (3.0), 63 (6.0), 65 (15.0), 77 (1.0), 89 (7.0), 90 (4.0), 91 (54.0), 105 (2.0), 119 (100.0), 120 (9.0), 135 (2.0), 150 (40.0)
M37	16.9	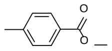	39 (6.0), 63 (7.0), 65 (16.0), 91 (47.0), 119 (100.0), 120 (7.0), 150 (33.0)<br>Standard: 39 (5.0), 63 (6.0), 65 (15.0), 91 (44.0), 119 (100.0), 120 (9.0), 150 (34.0)
M38	19.1<br>19.3 st	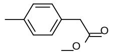	51 (4.0), 77 (13.0), 78 (6.0), 79 (9.0), 91 (3.0), 103 (9.0), 105 (100.0), 106 (9.0), 164 (29.0)<br>Standard: 51 (5.0), 77 (12.0), 78 (5.0), 79 (8.0), 91 (2.0), 103 (8.0), 105 (100.0), 106 (9,0), 164 (22.0)
M39	19.3	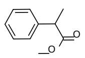	51 (4.0), 77 (11.0), 78 (4.0), 79 (8.0), 91 (2.0), 103 (7.0), 104 (5.0), 105 (100.0), 106 (8.0), 133 (1.0), 164 (27.0)<br>51, 77, 78, 79, 91, 103, 104, 105, 106, 133, 164 standard from the library NIST
M40	19.8<br>19.8 st	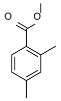	77 (17.0), 79 (4.0), 91 (6.0), 103 (14.0), 104 (7.0), 105 (50.0), 119 (6.0), 133 (100.0), 149 (22.0), 164 (48.0)<br>Standard: 77 (23.0), 79 (13.0), 91 (6.0), 103 (15.0), 104 (16.0), 105 (41.0), 119 (2.0), 133 (100.0), 149 (5.0), 164 (45.0)
M41	20.2	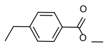	51 (6.0), 63 (4.0), 77 (16.0), 79 (11.0), 89 (7.0), 90 (5.0), 103 (11.0), 105 (37.0), 121 (6.0), 133 (100.0), 134 (9.0), 149 (9.0), 164 (32.0)<br>51, 63, 77, 79, 89, 90, 103, 105, 121, 133, 134, 149, 164 standard from the library NIST
M42	21.5	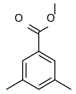	77 (15.0), 78 (7.0), 79 (9.0), 103 (13.0), 104 (3.0), 105 (29.0), 133 (100.0), 134 (9.0), 149 (2.0), 164 (31.0)<br>77, 78, 79, 103, 104, 105, 133, 134, 149, 164 standard from the library NIST
M43	21.8	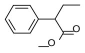	51 (5.0), 77 (16.0), 78 (9.0), 79 (9.0), 91 (15.0), 105 (100.0), 118 (67.0), 147 (9.0), 178 (35.0)<br>51, 77, 78, 79, 91, 105, 118, 147, 178 standard from the library NIST

Continuation Supplementary Table S13

No	t_R (min)	Metabolites	m/z (%) (70 eV) - analyzed with GC/MS
M 44	22.3	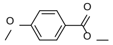	41 (5.0), 63 (9.0), 64 (8.0), 77 (20.0), 92 (15.0), 107 (10.0), 135 (100.0), 166 (34.0)<br>41, 63, 64, 77, 92, 107, 135, 166 standard from the library NIST
M45	22.6	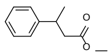	51 (5.0), 77 (16.0), 78 (9.0), 79 (9.0), 91 (15.0), 92 (8.0), 104 (43.0), 105 (100.0), 106 (11.0), 117 (23.0), 118 (67.0), 119 (13.0), 146 (16.0), 147 (9.0), 178 (35.0)<br>51, 77, 78, 79, 91, 92, 104, 105, 106, 117, 118, 119, 146, 147, 178 standard from the library NIST
M 46	29.2<br>29.1 st	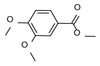	51 (15.0), 77 (25.0), 79 (39.0), 94 (14.0), 107 (14.0), 137 (21.0), 165 (100.0), 181 (14.0), 196 (88.0)<br>Standard: 51 (16.0), 77 (15.0), 79 (27.0), 94 (10.0), 107 (9.0), 137 (10.0), 165 (100.0), 181 (9.0), 196 (92.0)

(st: purchased standard; standard from the library NIST only shows the fragment without the intensity)

Supplementary Table S14. Mass spectrometric data of naphthylalkanoic and biphenylalkanoic acids identified during growth on crude oil of *M. spathulata* SBUG-Y 2180. These compounds were analysed by GC/MS. The extracted acids were transformed for analytical purposes by methylation to the corresponding methyl esters.

No	t_R (min)	Metabolites	m/z (%) (70 eV) - analyzed with GC/MS
M47	30.42	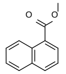	51 (5.0), 75 (15.0), 76 (5.0), 77(8.0), 87 (9.0), 101 (4.0), 126 (26.0), 127 (100.0), 155 (98.0), 186 (8.0)<br>51, 75, 76, 77, 87, 101, 126, 127, 155, 186 standard from the library NIST
M48	30.2	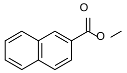	63 (16.0), 77 (14.0), 101 (7.0), 126 (21.0), 127 (84.0), 128 (15.0), 155 (100.0), 186 (63.0)<br>63, 77, 101, 126, 127, 128, 155, 186 standard from the library NIST
M49	32.4	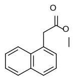	63 (5.0), 115 (29.0), 139 (20.0), 141 (100.0), 142 (8.0), 155 (1.0), 200 (40.0)<br>63, 115, 139, 141, 142, 155, 200 standard from the library NIST
M50	35.7<br>35.9 st	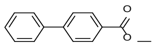	51 (5.0), 76 (23.0), 102 (3.0),126 (5.0), 127 (5.0), 151 (34.0), 152 (42.0), 153 (13.0), 181 (100.0), 182 (23.0), 212 (51.0)<br>Standard: 51 (4.0), 76 (20.0), 102 (3.0), 126 (5.0), 127 (5.0), 151 (16.0), 152 (58.0), 153 (30.0), 181 (100.0), 182 (14.0), 212 (61.0)
M51	37.6<br>37.7 st	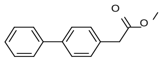	51 (2.0), 63 (6.0), 82 (4.0), 83 (4.0), 102 (1.0), 115 (7.0), 128 (18.0), 139 (6.0), 152 (27.0), 165 (29.0), 166 (9.0), 167 (100.0), 168 (13.0), 226 (52.0)<br>Standard: 51 (1.0), 63 (2.0), 82 (4.0), 83 (4.0), 102 (1.0), 115 (5.0), 128 (3.0), 139 (3.0), 152 (12.0), 165 (26.0), 166 (7.0), 167 (100.0), 168 (14.0), 226 (36.0)

(st: purchased standard; standard from the library NIST only shows the fragment without the intensity)

Supplementary Table S15. Mass spectrometric data of pristane transformation products identified during incubation of *M. spathulata* SBUG-Y 2180 with pristane and tetradecane. These compounds were analysed by GC/MS. The extracted acids were transformed for analytical purposes by methylation to the corresponding methyl esters.

No	t_R (min)	Metabolites	m/z (%) (70 eV) - analyzed with GC/MS
MP1	15.7<br>St. 16.3	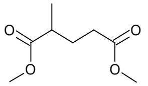<br>2-methylpentanedioic acid	39 (11.0), 41 (17.0), 42 (13.0), 43 (14.0), 45 (7.0), 55 (86.0) 56 (25.0), 59 (62.0), 69 (11.0), 73 (51.0), 74 (9.0), 83 (38.0), 87 (14.0), 88 (37.0), 99 (40.0), 101 (16.0), 114 (100.0), 115 (52.0), 142 (23.0), 143 (49.0), 174 (1.0)<br>Standard: 39 (11.0), 41 (17.0), 42 (12.0), 43 (14.0), 45 (6.0), 55 (74.0) 56 (22.0), 59 (54.0), 69 (8.0), 73 (51.0), 74 (9.0), 83 (33.0), 87 (12.0), 88 (29.0), 99 (36.0), 101 (12.0), 114 (100.0), 115 (48.0), 142 (19.0), 143 (49.0), 174 (1.0)
MP2	23.5	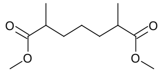<br>2,6-dimethylheptanedioic acid	55 (33.0), 56 (13.0), 57 (14.0), 59 (29.0), 67 (3.0), 69 (29.0), 73 (11.0), 87 (6.0), 88 (100.0), 89 (5.0), 97 (34.0), 101 (10.0), 114 (10.0), 124 (7.0), 125 (12.0), 129 (42.0), 152 (10.0), 156 (7.0), 157 (14.0), 185 (9.0), 216 (1.0)<br>55, 56, 57, 59, 67, 69, 73, 87, 88, 89, 97, 101, 114, 124, 125, 129, 152, 156, 157, 185, 216 (Pirnik et al. 1974)
MP3	29.9	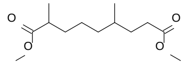<br>2,6-dimethylnonanedioic acid	55 (73.0), 59 (43.0), 69 (13.0), 73 (15.0), 74 (23.0), 83 (35.0), 87 (16.0), 88 (100.0), 97 (25.0), 98 (16.0), 101 (20.0), 115 (15.0), 124 (10.0), 125 (57.0), 126. (27.0), 139 (49.0), 157 (28.0), 180 (28.0), 185 (21.0), 213 (4.0)<br>structure deduced from fragmentation and retention time
MP4	39.2	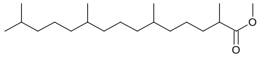<br>pristanic acid	41 (16.0), 43 (21.0), 55 (18.0), 56 (8.0), 57 (21.0), 69 (19.0), 71 (11,0), 88 (100,0), 97 (15.0), 101 (37.0), 222 (5.0), 312 (7.0)<br>41 (24.97), 43 (38.47), 55 (30.00), 56 (10.22), 57 (32.86), 69 (20.03), 71 (13.97), 88 (100.0), 97, 101 (37.56), 222, 312 (Pirnik et al. 1974; Nhi-Cong et al. 2009)
MP5	45.6	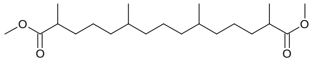<br>pristandioic acid	41 (20.0), 43 (10.0), 55 (45.0), 57 (23.0), 59 (19.0), 69 (40.0), 83 (24.0), 88 (100.0), 97 (42.0), 98 (31.0), 101 (20.0), 111 (6.0), 112 (11.0), 125 (17.0), 126 (95.0), 139 (7.0), 157 (4.0), 195 (5.0), 237 (7.0), 269 (17.0), 297 (5.0), 356 (1,0)<br>41, 43, 55, 57, 59, 69, 83, 88, 97, 98, 101, 111, 112, 125, 126, 139, 157, 195, 219, 237, 269, 297, 356 (Pirnik et al. 1974)

Supplementary Table S16. Mass spectrometric data of transformation products identified during incubation of *M. spathulata* SBUG-Y 2180 on biphenyl and on dibenzofuran. These compounds were analysed by HPLC/UV/Vis and GC/MS.

No	Metabolites	t_R HPLC (min)	UV/Vis max/min (nm)	t_R GC (min)	m/z (%) (70 eV) - analyzed with GC/MS
MBP1	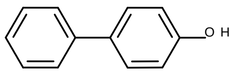	10.43	<210 (max), 228 (min), 254 (max),	13.46	39 (4.0), 51 (4.0), 55 (4.0), 63 (7.0), 85 (8.0), 89 (4.0), 115 (21.0), 139 (8.0), 141 (23.0), 169 (9.0), 170 (100.0)
	4-hydroxybiphenyl	St 10.39	St <210 (max), 228 (min), 254 (max),	St 13.48	St 39 (2.0), 51 (3.0), 55 (2.0), 63 (5.0), 85 (6.0), 89 (4.0), 115 (19.0), 139 (7.0), 141 (22.0), 169 (9.0), 170 (100.0)
MBP2	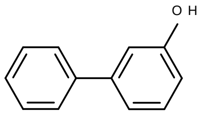		n.d.	13.35	39 (2.0), 51 (5.0), 63 (7.0), 115 (20.0), 139 (7.0), 141 (26.0), 169 (9.0), 170 (100.0)
	3-hydroxybiphenyl			St 13.37	St 39 (3.0), 51 (5.0), 63 (5.0), 115 (18.0), 139 (8.0), 141 (25.0), 169 (9.0), 170 (100.0)
MDBF1	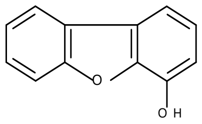		n.d.	14.18	51 (9.0), 63 (9.0), 77 (6.0), 92 (11.0), 102 (10.0), 126 (7.0), 127 (15.0), 128 (16.0), 155 (8.0), 184 (100.0)
	4-hydroxydibenzofuran				St 51, 63, 77, 92, 102, 126, 127, 128, 155, 184 standard from the library NIST
MDBF2	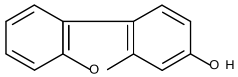	10.93	214 (max), 232 (max), 242 (min), 254 (max), 266 (min), 298 (max)	15.52	51 (8.0), 63 (9.0), 77 (7.0), 92 (14.0), 102 (9.0), 126 (7.0), 127 (14.0), 128 (23.0), 155 (12.0), 184 (100.0)
	3-hydroxydibenzofuran	St 11.00	St 214 (max), 232 (max), 242 (min), 254 (max), 266 (min), 298 (max)	St 15.51	St 51 (7.0), 63 (7.0), 77 (6.0), 92 (12.0), 102 (8.0), 126 (7.0), 127 (13.0), 128 (22.0), 155 (11.0), 184 (100.0)
MDBF3	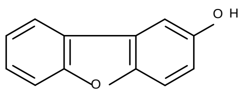	10.60	212 (max), 232 (mix), 242 (max), 248 (min), 252 (max), 262 (min), 290 (max), 304 (mix), 314 (max)	15.41	51 (9.0), 63 (9.0), 77 (7.0), 92 (13.0), 102 (11.0), 126 (8.0), 127 (16.0), 128 (24.0), 155 (11.0), 184 (100.0)
	2-hydroxydibenzofuran	St 10.62	St 212 (max), 232 (mix), 242 (max), 248 (min), 252 (max), 262 (min), 290 (max), 304 (mix), 314 (max)	St 15.39	St 51 (7.0), 63 (7.0), 77 (6.0), 92 (13.0), 102 (10.0), 126 (7.0), 127 (15.0), 128 (22.0), 155 (10.0), 184 (100.0)

Continuation Supplementary Table S16

MDBF4	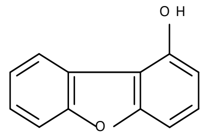	10.43	<210 (max), 220 (mix), 226 (max), 242 (mix), 256 (max), 266 (min), 278 (max), 290 (min), 298 (max)	15.26	51 (12.0), 63 (12.0), 77 (10.0), 92 (12.0), 102 (18.0), 126 (11.0), 127 (21.0), 128 (44.0), 155 (15.0), 184 (100.0)
	1-hydroxydibenzofuran	10.15	St <210 (max), 220 (mix), 226 (max), 242 (mix), 256 (max), 266 (min), 278 (max), 290 (min), 298 (max)		St 51, 63, 77, 92, 102, 126, 127, 128, 155, 184 standard from the library NIST

n.d. not found using this method

Supplementary Table S17. Mass spectrometric data of products identified during incubation of *M. spathulata* SBUG-Y 2180 on *n*-undecylcyclohexane. These compounds were analysed by GC/MS. The extracted acids were transformed for analytical purposes by methylation to the corresponding methyl esters.

No	t_R (min)	Metabolites	m/z (%) (70 eV) - analyzed with GC/MS
$MC_6C_{11}1$	11.6<br>11.6 st	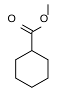	41 (39.0), 55 (100.0), 67 (22.0), 68 (18.0), 69 (12.0), 74 (37.0), 81 (17.0), 82 (27.0), 83 (76.0), 87 (90.0), 101 (15.0), 110 (27.0), 111 (21.0), 113 (22.0), 127 (8.0), 142 (31.0)<br>Standard: 41 (32.0), 55 (92.0), 67 (21.0), 68 (19.0), 69 (13.0), 74 (39.0), 81 (17.0), 82 (28.0), 83 (81.0), 87 (100.0), 101 (17.0), 110 (31.0), 111 (25.0), 113 (26.0), 127 (10.0), 142 (40)
$MC_6C_{11}2$	12.8<br>12.9 st	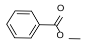	50 (9.0), 51 (20.0), 77 (60.0), 91 (2.0), 92 (3.0), 105 (100.0), 106 (8.0), 136 (33.0)<br>Standard: 50 (7.0), 51 (17.0), 77 (56.0), 91 (2.0), 92 (2.0), 105 (100.0), 106 (8.0), 136 (38.0)
$MC_6C_{11}3$	14.3<br>14.3 st	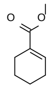	39 (13.0), 41 (12.0), 51 (9.0), 52 (6.0), 53 (22.0), 65 (6.0), 77 (19.0), 79 (61.0), 80 (66.0), 81 (100.0), 108 (23.0), 109 (25.0), 140 (36.0)<br>Standard: 39 (14.0), 41 (14.0), 51 (10.0), 52 (7.0), 53 (25.0), 65 (6.0), 77 (19.0), 79 (66.0), 80 (71.0), 81 (100.0), 108 (25.0), 109 (26.0), 140 (35.0)
$MC_6C_{11}4$	14.8<br>14.8 st	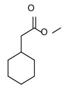	41 (12.0), 43 (20.0), 55 (20.0), 67 (8.0), 74 (100.0), 75 (33.0), 81 (6.0), 97 (5.0), 113 (2.0), 125 (8.0), 156 (1.0)<br>Standard: 41 (9.0), 43 (15.0), 55 (16.0), 67 (7.0), 74 (100.0), 75 (34.0), 81 (6.0), 97 (6.0), 113 (2.0), 125 (9.0), 156 (1.0)
$MC_6C_{11}5$	15.7<br>15.8 st	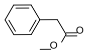	39 (3.0), 51 (3.0), 59 (6.0), 65 (12.0), 77 (1.0), 89 (5.0), 91 (100.0), 92 (6.0), 105 (1.0), 119 (4.0), 150 (23.0)<br>Standard: 39 (4.0), 51 (2.0), 59 (5.0), 65 (12.0), 77 (1.0), 89 (5.0), 91 (100.0), 92 (8.0), 105 (1.0), 119 (2.0), 150 (34.0)
